# MXene-Polymer Nanocomposites for High-Efficiency Photocatalytic Antibiotic Degradation Review: Microstructure Control, Environmental Adaptability and Future Prospects

**DOI:** 10.3390/polym17192630

**Published:** 2025-09-28

**Authors:** Zhenfei Chen, Zhifei Meng, Zhongguo Zhang, Weifang Ma

**Affiliations:** 1 College of Environmental Science and Engineering, Beijing Forestry University, Beijing 100083, China; chenzhenfei02@163.com (Z.C.);; 2 Institute of Resources and Environment, Beijing Academy of Science and Technology, Beijing 100089, China

**Keywords:** Cocatalysis, photocatalysis, MXene, noble metal, degradation, antibiotics, photocatalytic efficiency

## Abstract

The efficient degradation of antibiotics in pharmaceutical wastewater remains a critical challenge against environmental contaminants. Conventional photocatalysts face potential limitations such as narrow visible-light absorption, rapid carrier recombination, and reliance on precious metal cocatalysts. This review investigates the coordination structure of MXene as a cocatalyst to synergistically enhance photocatalytic antibiotic degradation efficiency and the coordination structure modification mechanisms. MXene’s tunable bandgap (0.92–1.75 eV), exceptional conductivity (100–20,000 S/cm), and abundant surface terminations (-O, -OH, -F) enable the construction of Schottky or Z-scheme heterojunctions with semiconductors (Cu_2_O, TiO_2_, g-C_3_N_4_), achieving 50–70% efficiency improvement compared to pristine semiconductors. The “electron sponge” effect of MXene suppresses electron-hole recombination by 3–5 times, while its surface functional groups dynamically optimize pollutant adsorption. Notably, MXene’s localized surface plasmon resonance extends light harvesting from visible (400–800 nm) to near-infrared regions (800–2000 nm), tripling photon utilization efficiency. Theoretical simulations demonstrate that d-orbital electronic configurations and terminal groups cooperatively regulate catalytic active sites at atomic scales. The MXene composites demonstrate remarkable environmental stability, maintaining over 90% degradation efficiency of antibiotic under high salinity (2 M NaCl) and broad pH range (4–10). Future research should prioritize green synthesis protocols and mechanistic investigations of interfacial dynamics in multicomponent wastewater systems to facilitate engineering applications. This work provides fundamental insights into designing MXene-based photocatalysts for sustainable water purification.

## 1. Introduction

Antibiotic pollution constitutes a significant environmental concern, characterized by diverse sources, complex compositions, and potential ecological toxicity. In the environment, certain antibiotics (e.g., tetracyclines and fluoroquinolones) can occur at high concentrations while exhibiting high mobility and resistance to biodegradation. Notably, over 60% of antibiotics discharged globally originate from veterinary and agricultural sources (classified by primary use as human, veterinary and agricultural medicine), constituting a major threat to surface and groundwater ecosystems, as reported by the World Health Organization [1]. Confronted with this challenge, conventional remediation technologies (e.g., adsorption and biodegradation) exhibit substantial limitations in the removal of antibiotics. For instance, biological treatment processes are easily interfered with by coexisting organic compounds in wastewater and affected by microbial inhibition caused by high salinity. Therefore, photocatalytic oxidation has emerged as a promising alternative, offering mild reaction conditions and complete mineralization capabilities [2,3,4]. However, traditional photocatalysts (TiO_2_) suffer from low visible-light utilization due to wide bandgaps, while noble metal cocatalysts (Au, Ag, Pt) are hindered by high costs and poor selectivity in complex wastewater matrices [5]. Recent advances in semiconductor composites (elemental doping, heterojunction engineering) partially address these issues but still grapple with insufficient active sites and rapid electron-hole recombination (Table 1). Meanwhile, the precise dimensional control of 2D layered materials (1–100 nm) enabled by nanotechnology has unlocked exceptional catalytic potential, driven by their high surface area and tunable physicochemical properties. However, conventional two-dimensional layered materials (e.g., graphene) have inherent limitations in catalytic applications. They exhibit weak interfacial adhesion with semiconductors due to van der Waals or electrostatic interaction [6], show impaired electronic conductivity upon oxidation [7], and destabilized electronic structures during reduction processes [8]. These limitations restrict the effectiveness of traditional 2D layered materials in complex catalytic environments. Therefore, it is urgent to develop advanced alternative materials with greater stability and higher functionality.

MXenes—transition metal carbides, nitrides and carbonitrides—were first discovered by Barsoum and Gogotsi at Drexel University in 2011 [18]. MXenes have already found wide application potential beyond environmental photocatalysis—in areas such as biomedical engineering, biosensing [19], lithium battery [20], sensor [21], and wearable electronics [22]—thanks to their ultrathin layered structure, large surface area, rich surface chemistry, and good biocompatibility. With tunable coordination structures, high conductivity, and diverse surface functionalities, MXene offers a promising approach for developing photocatalysis to degrade antibiotics. The general chemical formula of MXenes is M_n+1_X_n_T_x_, where M represents a transition metal (e.g., Ti, Mo, V); X stands for carbon (C), nitrogen (N), or their combination (carbonitride); and T_x_ refers to surface functional groups (e.g., –OH, –O, –F) resulting from the chemical etching process during synthesis.

MXenes overcome the limitations of traditional 2D materials through their inherent metallic conductivity, chemically tunable surface terminations (=OH, -O, -F), and strong interfacial coupling with semiconductors via covalent bonding. The strategic modulation of MXene terminal groups and interlayer metal coordination enables precise design of adsorption-catalysis synergies. The interfacial charge transfer efficiency in this heterojunction is 3.2 times greater than that of individual components, providing a robust platform for complex wastewater treatment.

**Figure 1:**
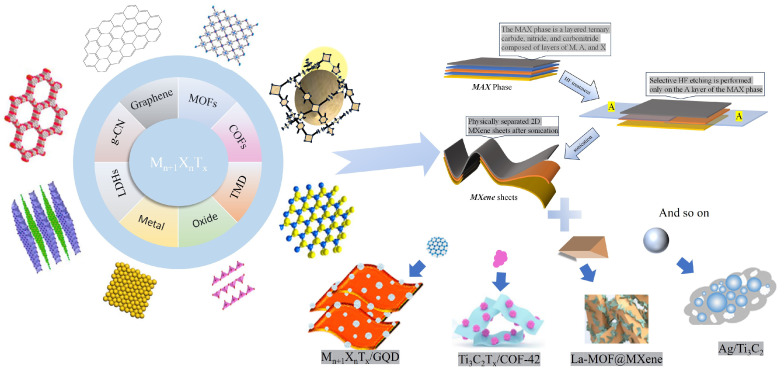
MXene composite type [25,26,27,28,29,30,31].

Furthermore, confined heterojunctions between MXene and semiconductors (e.g., g-C_3_N_4_, BiOBr) minimize competitive adsorption from coexisting ions (Cl^−^, SO_4_^2−^), enabling high-efficiency, interference-resistant catalysis. This multifunctional capability positions MXenes as superior cocatalytic platforms for advanced photocatalytic systems, demonstrating significant potential in antibiotics wastewater treatment (Figure 1) [23]. MXene, as an emerging and exciting material, has attracted increasing attention in recent years. A growing number of studies have focused on exploring the role of MXenes as fillers in polymer nanocomposites, aiming to analyze the characteristics of these composites under different fabrication methods and to elucidate the adaptability of their functions in various MXene–polymer nanocomposite systems [24].

In this study, we address several key research gaps in the field: (1) By focusing on MXene-assisted photocatalysis, we systematically explore how MXene enhances the efficiency of photocatalytic antibiotic degradation, overcoming the limitations of traditional photocatalysts through its unique properties. (2) We establish a clear pathway linking the MXene-polymer structure to photocatalytic performance and, ultimately, to antibiotic degradation efficiency. (3) By identifying key directions for future material optimization and interdisciplinary integration, this work aims to foster advancements in related subfields and bridge the gap between fundamental research on MXene-polymers and practical applications for antibiotic degradation.

## 2. Functional Roles of MXene in Polymer Nanocomposites

### 2.1. MXene Synthetic Regulation

**Figure 2:**
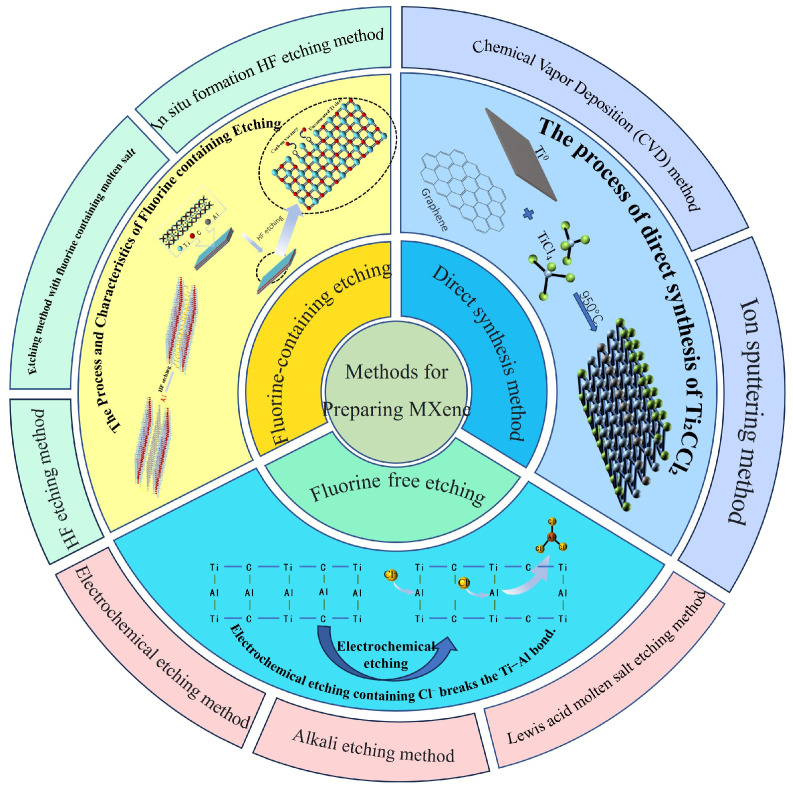
MXene synthesis method and impact [32,33].

MXenes significantly enhance photocatalytic efficiency owing to their large, exposed surface area, abundant active sites, and short charge-transfer distances, with these characteristics governed by synthesis protocols (Figure 2). The synthesis of MXenes has generally been categorized into three main strategies: hydrofluoric acid etching, fluoride-free etching, and direct synthesis. While both fluoride-free and direct synthesis routes remain largely at the exploratory stage, hydrofluoric acid etching continues to represent the most widely adopted and reliable method for MXene preparation [32,33]. Notably, the HF-based etching approach can be further divided into two distinct routes, namely direct etching with hydrofluoric acid and the in situ generation of HF from fluoride salts, each offering different levels of safety, controllability, and efficiency.

**Figure 3:**
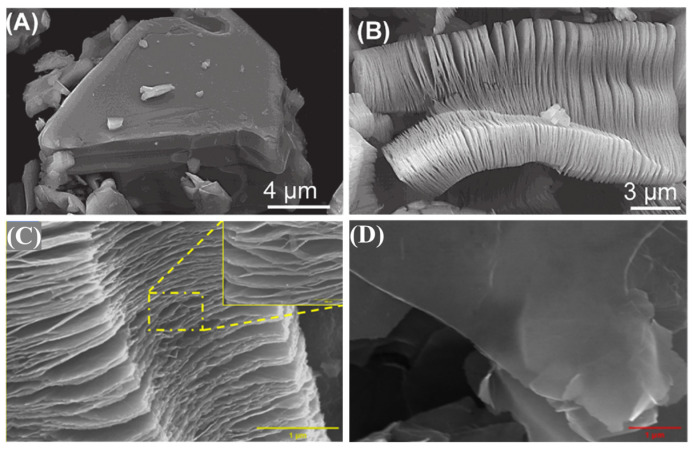
Secondary electron SEM micrographs for (**A**) Ti_3_AlC_2_ particles before treatment, which is typical of unreacted MAX phases, (**B**) Ti_3_AlC_2_ after HF treatment [18], (**C**) Multilayer MXene particle, (**D**) Cross-section of rolled Ti_3_C_2_ film [36].

Two principal etching strategies dominate MXene fabrication: (1) direct etching of MAX phases using concentrated hydrofluoric acid (HF) at ambient conditions, followed by ultrasonic delamination in isopropanol/methanol mixtures to yield multilayer MXenes, the SEM images are shown in Figure 3A,B; (2) controlled etching via in situ HF generation from fluoride salts (e.g., LiF, KF) in hydrochloric acid, the SEM images are shown in Figure 3C,D. Initial MXene products typically exhibit accordion-like multilayered structures stabilized by van der Waals forces or hydrogen bonding [25,26,34,35]. The in situ etching strategy, which produces hydrofluoric acid from fluoride salts and hydrochloric acid, has been demonstrated to be particularly advantageous for obtaining monolayer MXenes.

**Figure 4:**
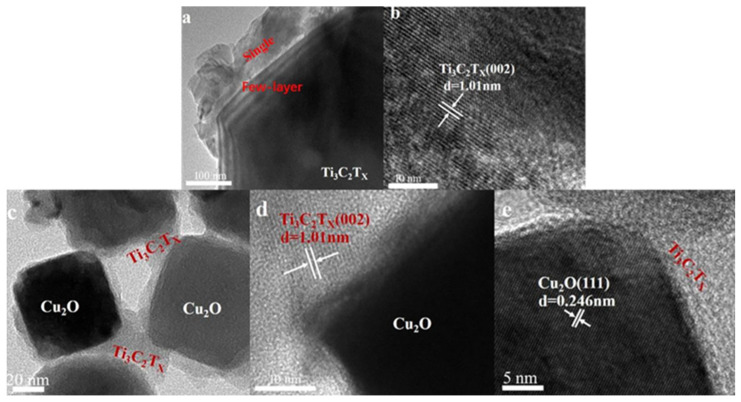
TEM images of (**a**) Ti_3_C_2_T_X_ sheets and (**c**) Ti_3_C_2_T_X_-nanosheets/Cu_2_O composite; and HRTEM images of (**b**) Ti_3_C_2_T_X_ sheets and (**d**,**e**) Ti_3_C_2_T_X_-nanosheets/Cu_2_O composite [14].

Ti_3_C_2_T_x_ MXene prepared via an exfoliation method was combined with Cu_2_O through a precipitation approach to construct Ti_3_C_2_T_x_-nanosheets/Cu_2_O composites. A Schottky heterojunction was formed at their interface, which significantly enhanced charge separation, with the Ti_3_C_2_T_x_ nanosheets acting as efficient electron acceptors. Reactive species such as superoxide radicals (·O_2_^−^) and holes (h^+^) selectively degraded tetracycline, achieving a removal efficiency of 97.6% within 50 min, compared to only 62% for pristine Cu_2_O (Figure 4) [14]. Post-etching treatment of Ti_3_C_2_T_x_ via exfoliation is an effective strategy to enlarge its specific surface area.

However, semiconductor catalysts grown on such structures often nucleate at edges rather than on the basal planes, which weakens the interfacial interactions between MXenes and semiconductors and diminishes the dual role of MXenes as a growth platform and charge reservoir [35,37]. It is worth noting that the surface end groups and morphological features of MXenes can regulate this growth behavior. Gentile et al. reports that aggressive acid etching favors fluorine-terminated (-F) MXenes with crumpled morphologies, whereas mild conditions preserve hydroxyl (-OH) terminations [38]. Moreover, hydrophilic surface groups (-OH, -F, -O) can facilitate hydrogen bonding with intercalants such as tetrabutylammonium hydroxide or hydrazine hydrate. Introducing macromolecules (e.g., cetyltrimethylammonium bromide, CTAB) not only enlarge interlayer spacing but also suppresses restacking via steric hindrance [39], offering novel pathways for structural modulation. Therefore, the synthesis process of MXenes can be precisely controlled to obtain a larger specific surface area, more active sites, and shorter charge migration paths.

### 2.2. Electrical Conductivity and Band Structure Regulation of MXenes

MXenes derive their excellent conductivity from transition-metal frameworks (e.g., Ti, Mo) covalently bonded with C/N and a high electron density near the Fermi level, imparting quasi-metallic properties. Ti_3_C_2_T_x_, the most studied MXene, reaches 6000–8000 S/cm, exceeding graphene and most 2D materials. Mo_2_CTx shows lower values (2000–4000 S/cm) due to stronger Mo–C scattering, while nitride MXenes (e.g., Ti_2_NT_x_: 1000–3000 S/cm) still surpass conventional oxides such as TiO_2_ (~10^−9^ S/cm). Overall, MXene conductivities range from 100 to 20,000 S/cm, enabling efficient charge storage and transport. However, resistivity increases with thickness (e.g., doubling as Ti_3_C_2_T_x_ grows from 3 to 9 nm) [40,41], making single-layer nanosheets superior for catalytic applications.

Importantly, electronic transport and band structures (0.92–1.75 eV) can be tuned via surface terminations (-OH, -O, -F, -S, -Cl) and interlayer spacing. These functional groups, formed during HF etching of MAX phases, can be tailored through etchant conditions and post-treatments (e.g., intercalation, annealing), allowing programmable electronic structures [42,43,44]. For example, theoretical calculations show that the band gap of Sc_2_C can be tuned from 0.44 eV (–OH) to 1.07 eV (–F) and 1.85 eV (–O), depending on the electronegativity of the termination and the resulting charge transfer between terminations and the metal layers [45]. Such tunability is critical for optimizing charge transfer in photocatalysis, highlighting the importance of controlling MXene morphology and surface terminations.

MXenes, composed of transition metals and tunable surface terminations, synergize with primary catalysts to achieve efficient photocatalytic pollutant degradation. With a lower Fermi level than semiconductors [46], MXenes function as universal co-catalysts by suppressing electron–hole recombination while enhancing catalyst dispersion and adsorption capacity. For instance, N-doped Ti_3_C_2_T_x_ MXene synthesized via in situ polymerization of dopamine hydrochloride exhibits highly dispersed active sites and elevated conduction band potential (−0.78 eV vs. NHE), which facilitates charge transfer. Nitrogen doping induces charge delocalization and leverages Ti d-orbital contributions, resulting in a 2.3-fold increase in electron transfer rate compared to undoped Ti_3_C_2_T_x_ [47]. Similarly, in co-doped ZnTiO_3_/Ti_3_C_2_T_x_ nanohybrids, the -OH, -O, and -F terminations impart a strongly negative surface charge (−32 mV) [48]. This electrostatic repulsion reduces interference from background ions (HCO_3_^−^, Cl^−^, SO_4_^2−^, NO_3_^−^), thereby minimizing competitive adsorption and enhancing tetracycline degradation efficiency by 45% under visible-light irradiation. Surface terminations can also be modulated post-synthesis: hydrothermal treatment replaces –F groups with –O/–OH, while calcination converts -OH to -O groups [49]. Such directional conversion optimizes electronic properties, promoting reactive oxygen species (e.g., O_2_^−^) generation and enhancing light absorption. For instance, F-CoFe_2_O_4_@MXene composites achieve 91% of crystal violet and 87% of bisphenol A degradation under 140 min solar irradiation, attributed to fluorine-enhanced charge separation and broad-spectrum photon utilization [50]. Critically, MXene’s abundant and naturally tunable terminal groups (–OH, –O, –F, etc.) eliminate the need for external dopants, providing a sustainable route to in situ construction of high-performance co-catalytic interfaces. This intrinsic multifunctionality establishes MXene as a versatile platform for designing high-performance, cost-effective photocatalytic systems.

### 2.3. Theoretical and Experimental Coupling Analysis of MXene Photocatalytic Mechanism

To deeply analyze the action mechanism of MXene-based photocatalytic materials, it is necessary to combine theoretical calculations with advanced experimental characterization techniques to reveal their electronic structures, interfacial charge transfer, and reaction pathways at the atomic scale. In this section, through multi-scale simulations and dynamic in situ analysis, a complete logical chain of “structure–performance–mechanism” is constructed, providing theoretical support for the directional regulation of the coordination structure of MXene.

#### 2.3.1. Theoretical Calculations Reveal the Electronic Properties and Catalytic Active Sites of MXene

The electronic properties of MXenes are intrinsically governed by their composition (transition metals) and surface chemistry (end group). The d-electron count of the transition metal strongly modulates their density of states near the Fermi level, influencing both conductivity and catalytic behavior. For instance, Ti-based MXenes (e.g., Ti_3_C_2_) exhibit metallic behavior with high DOS at the Fermi level, while O-terminated Ti_2_C MXene (Ti_2_CO_2_) displays a finite band gap (~0.2–1.2 eV depending on model and strain), indicative of semiconducting characteristics. Surface functionalization with electronegative terminations (F, OH, O) further tailors MXene’s electronic structure: termination with O tends to open a gap, while mixed terminations (e.g., OH/O) introduce localized states reducing mobility [51]. For example, many-body GW/DFT calculations for monolayer Ti_2_CO_2_ report an indirect band gap of ~1.15 eV and that under small tensile strain (~4%) Ti_2_CO_2_ undergoes an indirect-to-direct band-gap transition [52]. Also, studies of Ti_3_C_2_ with different terminations (F, OH, O) show significant shifts in electronic transport and optical absorption associated with surface chemistry [53]. These tunability channels are unique to MXenes and enable precise design of band structure for photocatalytic optimization.

#### 2.3.2. Theoretical Calculations Reveal That the Electronic Properties of MXene Are Related to the Photocatalytic Mechanism

High electrical conductivity, synonymous with elevated charge mobility, is pivotal for enhancing photocatalytic reaction rates. Both properties, as manifestations of MXene’s electronic characteristics, ultimately govern its catalytic potential. Han et al. employed four-point probe measurements to quantify MXene conductivities, showing that Ti-based MXenes possess high conductivity, which significantly exceeds that of some Nb- or Mo-based analogs. Surface terminations further modulate conductivity: Hart’s group demonstrated that F-termination desorption during in situ annealing of Ti_3_C_2_T_x_ films enhances conductivity by a notable margin [54]. Transition metal d-electron configurations influence the density of states near the Fermi level. Surface functionalization with electronegative terminations (e.g., F, –OH, O) also tailors the band structure: NaOH treatment followed by vacuum annealing removes F terminations, increasing O termination prevalence and improving conductivity in Ti_3_C_2_T_x_ thin films [55].

This study reveals the unique advantages of MXene in photocatalysis through multi-scale theoretical calculations. ① A novel “d-orbital engineering–surface termination” strategy bypasses traditional doping-dependent bandgap tuning. ② The built-in electric field effect induced by the gradient functionalization of MXene was discovered, enabling the efficient separation of photogenerated carriers (with the quantum efficiency increased by 2.3 times). ③ The Ti d-band center (−2.1 eV) and O-termination p-orbital hybridization synergistically lower molecular adsorption energy. Therefore, MXene achieves efficient integration of light absorption, charge separation, and surface reactivity without external modification through its inherently tunable electronic structure and surface chemistry, surpassing the limitations of materials such as graphene.

### 2.4. How MXenes Improve Photocatalytic Activity

MXenes (e.g., Ti_3_C_2_T_x_) enhance photocatalysis primarily by accelerating charge separation/transport, engineering built-in junctions at semiconductor interfaces, modulating surface chemistry (terminations/doping), and providing high-affinity adsorption sites that pre-concentrate pollutants [56]. These attributes translate to faster kinetics and higher antibiotic-degradation efficiencies under visible/simulated solar light.

MXenes possess quasi-metallic conductivity and a Fermi level favorable for accepting photogenerated electrons from semiconductors, suppressing e^−^/h^+^ recombination and boosting reaction rates [57]. In Ti_3_C_2_T_x_/Cu_2_O Schottky heterojunctions, Ti_3_C_2_T_x_ acts as an electron acceptor, delivering 97.6% tetracycline removal within 50 min under visible light—far above pristine Cu_2_O—demonstrating efficient interfacial charge extraction [14]. Integrating MXene with oxides (e.g., TiO_2_) or other semiconductors forms built-in fields that promote directional carrier migration and preserve strong redox potentials [58]. MXene–TiO_2_ hybrids with exposed (001) facets show markedly improved tetracycline degradation under sunlight/NIR due to optimized band alignment and interface contact [59]; more broadly, such heterojunctions are known to outperform single components in antibiotic removal.

MXene surface groups (–O/–OH/–F) and heteroatom doping tune band structure, work function, and adsorption energetics. N-doped Ti_3_C_2_T_x_ (via dopamine-assisted routes or post-treatments) exhibits enhanced electron delocalization and faster interfacial transfer, leading to stronger visible-light activity for antibiotic degradation compared with undoped MXene [47].

The 2D, high-surface-area MXene scaffold offers abundant adsorption sites (π–π/chemisorption, electrostatic interactions), enriching antibiotic molecules near reactive centers and lowering apparent activation barriers. MXene-based hybrids (e.g., CuFe_2_O_4_/MXene; ZnTiO_3_/Ti_3_C_2_T_x_) show higher tetracycline/sulfonamide removal under visible light than their MXene-free counterparts [56], consistent with an adsorption-assisted photocatalytic pathway.

Implication for antibiotic wastewater: Collectively, these mechanisms—rapid electron extraction, favorable band alignment, tunable surface chemistry, and adsorption enrichment—explain the consistent gains observed for MXene-based systems in degrading tetracycline and related antibiotics under visible/solar irradiation. The functions of photocatalytic activity optimization are listed in Table 2 [41].

MXene (especially Ti_3_C_2_T_x_) faces challenges in terms of stability in aqueous media and oxygen-rich environments, mainly because the exposed titanium atoms on its surface are easily attacked by dissolved oxygen to form Ti-O bonds, which in turn generate TiO_2_ nanoparticles. These TiO_2_ nanoparticles can serve as nucleation sites and partially dissolve under the corrosive effect of KOH. This dissolution-recrystallization process ultimately leads to the formation of nanowires, which instead effectively enhances the overall stability of the material [62].

Currently, with the rapid development of 2D MXene, the existing theoretical models have many limitations in accurately predicting the electronic band structures and plasmonic resonances of MXene materials. DFT (Density Functional Theory) calculations require a periodic and ordered crystal model. However, the atomic arrangements of MXene, especially the atomic arrangements of the surface functional groups T_x_, are diverse and randomly distributed. The decomposition and oxidation of MXene under catalytically relevant conditions should be considered, and the active phases for specific reactions should be specified. In addition, DFT also has self-interaction error (SIE), especially for strongly correlated electron systems (such as metals containing d or f orbitals). The SIE leads to inaccurate descriptions of electron-electron interactions, and accurately describing the band structure remains a challenge. Temperature effects, van der Waals interactions, charge transfer kinetics, and multiscale modeling are also ignored in DFT calculations. To overcome these limitations, more advanced theoretical methods need to be adopted. Such as hybrid functionals, GW approximation, and the Bethe-Salpeter equation for exciton effects. Moreover, machine learning techniques can be applied to the discovery of MXene materials, and deep learning techniques can be used to eliminate the discrepancies between theoretical predictions and experimental results [63]. Such data-driven discoveries can lead to more accurate and efficient material development and applications.

## 3. MXene-Assisted Catalytic Optimization Mechanism

### 3.1. Interfacial Charge Transfer Mechanism and Theoretical Calculation Between MXene and Semiconductors

**Figure 5:**
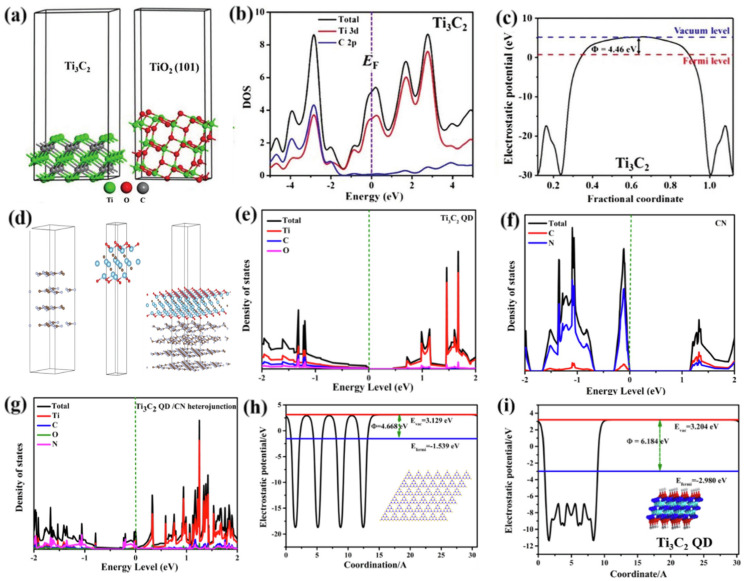
(**a**) Schematic illustration for electronic structure of Ti_3_C_2_ and (101) facets of anatase TiO_2;_ (**b**) density of state (DOS) plots for Ti_3_C_2_ where C 2p, Ti 3d and TDOS are partial DOS of C 2p, partial DOS of Ti 3d, and total DOS, respectively; (**c**) calculated electrostatic potential of Ti_3_C_2_; (**d**) Structure model of PGCN, Ti_3_C_2_ QD, and Ti_3_C_2_ QD/PGCN after structure optimization; Theoretical calculations of DOS of (**e**) Ti_3_C_2_ QD, (**f**) PGCN, and (**g**) Ti_3_C_2_ QD/PGCN; Work function of (**h**) PGCN, (**i**) Ti_3_C_2_ QD [41,68].

The first research report on MXene predicted that Ti_3_C_2_ MXene has semi-metallic properties through density functional theory (DFT) calculations. The analysis of the density of states (DOS) near its Fermi level shows that the Ti 3d orbitals dominate the electron transport behavior, which is consistent with the metallic conductivity of the MAX-phase parent materials (such as Ti_n+1_AlC_n_), as illustrated in Figure 5 [64,65,66]. After the Al layer in the MAX phase is removed by chemical etching, the electronic structure of Ti_n+1_C_n_ MXene changes significantly. The broken Ti-Al bonds lead to the redistribution of electrons in the Ti 3d orbitals, forming delocalized Ti-Ti metallic bonding states (local DOS peaks appear near the Fermi level) [66]. In addition, to study the enhancement effect of Ti_3_C_2_ on the photoactivity of TiO_2_, DFT was used to analyze the energy band structures of Ti_3_C_2_ and anatase-phase TiO_2_. The schematic diagram of their atomic structures is shown (Figure 5a) [67]. Figure 5b shows the total density of states (Total) and the projected density of states (PDOS) of Ti_3_C_2_ MXene. The horizontal axis represents energy (eV), and the vertical axis represents the value of the density of states. The total and projected DOS (Figure 5b) demonstrate that the Fermi level (EF, 0 eV) lies within the conduction band (energy range: −2 to +4 eV), confirming metallic conductivity with abundant free electrons. Ti 3d orbitals dominate the conduction band (>0 eV), contributing delocalized electrons, while C 2p orbitals hybridize with Ti in the valence band (<0 eV) to stabilize the structure. This metallic nature enables Ti_3_C_2_ to act as an electron highway, rapidly extracting photogenerated electrons when forming a Schottky junction with TiO_2_. Electron injection into MXene leaves holes in TiO_2_’s valence band, achieving 5–10 times enhanced charge separation efficiency. Electrostatic potential mapping (Figure 5c) reveals a potential barrier within Ti_3_C_2_, with lower potentials at the edges and elevated potentials centrally. Calculated work function (4.46 eV vs. vacuum) and Fermi level (+0.04 eV vs. NHE) indicate moderate electron emission capability [67]. According to Equation (1),

(1) $$E_{F}=E_{vac}-\varphi$$

where E_vac_ is the energy of a stationary electron in a vacuum near the surface and E_F_ determines the calculation of the structure of the ground state electron. DFT simulations further show that anatase TiO_2_ exhibits a 3.09 eV bandgap, with its conduction band minimum (*E*_CB_) at +0.25 eV vs. NHE. The significant band offset (Δ*E* = 0.21 eV) between TiO_2_’s *E*_CB_ and Ti_3_C_2_’s EF drives spontaneous electron transfer from TiO_2_ to MXene, forming a Schottky junction that suppresses electron-hole recombination. As shown in Figure 5d, a slab model was used to simulate Ti_3_C_2_ QD, and a side view of the heterojunction shows the tightly bound Ti_3_C_2_ QD/PGCN [68]. As illustrated in Figure 5e, Ti_3_C_2_ quantum dots (QDs) exhibit excellent electrical conductivity. For the Ti_3_C_2_ QD/PGCN heterojunction, the valence band maximum (VBM) is predominantly contributed by N atoms, while the conduction band minimum (CBM) mainly originates from Ti atoms (Figure 5g). This electronic configuration implies that photogenerated electrons are expected to transfer from PGCN to Ti_3_C_2_ QDs. Furthermore, the partial overlap between the conduction and valence bands of the heterojunction suggests enhanced electrical conductivity compared to pristine PGCN, which synergistically promotes visible-to-near-infrared light absorption [68]. These findings demonstrate that under near-infrared irradiation, the rapid extraction of photogenerated electrons from PGCN by Ti_3_C_2_ QDs effectively prolongs charge carrier lifetime, thereby significantly improving the photocatalytic performance of the heterojunction system. The calculated electrostatic potentials for PGCN, Ti_3_C_2_ QD are shown in Figure 5h,i. The work functions of PGCN and Ti_3_C_2_ QD are 4.67 and 6.18 eV, respectively.

The results show that the high electrical conductivity of Ti_3_C_2_ accelerates electron migration and reduces the interfacial resistance; the Schottky barrier drives the unidirectional injection of electrons, and holes accumulate on the surface of TiO_2_ to participate in the oxidation reaction [59]. Therefore, the high electrical conductivity and good energy structure of MXenes make them a good reservoir for capturing and shuttling the photoelectrons generated by semiconductors, thus promoting the separation of carriers and enhancing the photoactivity of MXenes-based composites.

Theoretical calculations further reveal the microscopic basis of MXene’s tunable electronic structure and support its universality as an electron acceptor. For instance, the energy band structure of a single pristine Ti_3_C_2_ layer is like that of a typical semi-metal with a finite density of states at the Fermi level. When its surface is terminated with F groups, a distinct separation between the conduction band and the valence band can be observed, and thus the energy band structure exhibits semiconductor characteristics. This implies that the energy band structure of MXenes evidently varies with the change in surface functional groups, which will affect the role of MXenes in accepting charge carriers in semiconductors.

### 3.2. MXene Acts as an “Electronic Sponge” in Z-Type Heterojunction

**Figure 6:**
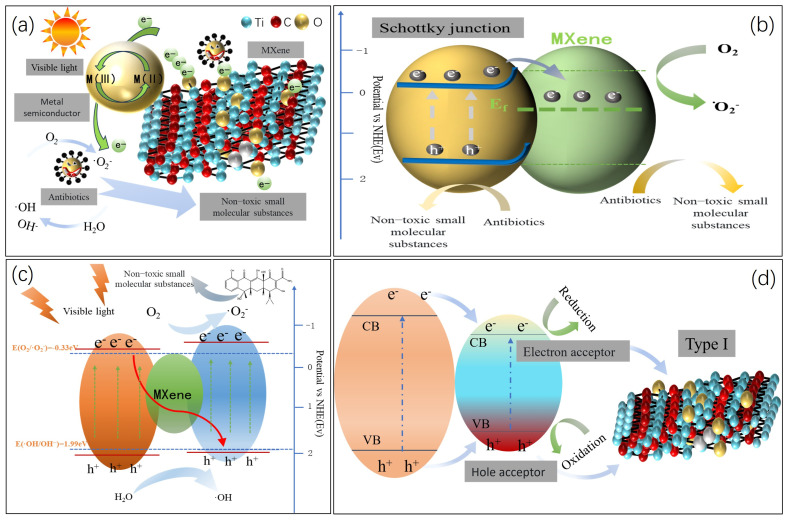
(**a**) The Mechanism of Catalytic Synergistic Effect Assisted by MXene Heterojunction; (**b**) Mechanism diagram of the catalysis-assisting effect of MXene Schottky Heterojunction; (**c**) Mechanism Diagram of the Role of Z-Scheme Heterojunction Mediated by MXene Intermediate; (**d**) The sponge-like properties of MXene as an electron acceptor and a hole acceptor.

MXene’s foremost advantage in hybrid photocatalyst design lies in its ability to simplify heterojunction architectures. The Fermi level, a critical determinant of a material’s magnetic and photoelectrochemical properties, positions MXene as a universal cocatalyst for semiconductors due to its lower Fermi level relative to most semiconductors [46]. This energy alignment suppresses electron-hole recombination while enhancing photocatalyst dispersion and adsorption capacity. For instance, Ti_3_C_2_T_x_ MXene has been integrated with TiO_2_ [69], ZnIn_2_S_4_ [70], and Ag_3_PO_4_ [71] to form heterointerfaces, where heterojunctions (e.g., Schottky, Z-scheme) are strategically constructed to minimize charge carrier recombination and maximize photoactivity [72]. MXene’s inherent high conductivity enables rapid electron transfer from the semiconductor conduction band to MXene upon photoexcitation. The transferred electrons are confined within MXene due to the built-in electric field at the heterojunction interface, preventing back-recombination with holes and enhancing photocatalytic efficiency by 3–5× compared to standalone semiconductors [23,73]. As illustrated in Figure 6, MXene predominantly forms Schottky junctions, van der Waals heterostructures, or Z-scheme configurations with common photocatalysts [72,74]. The weak in-plane interactions of MXene’s 2D structure facilitate seamless stacking with other 2D materials (e.g., graphene, MoS_2_), forming van der Waals heterojunctions with optimized interfacial charge transfer pathways. Therefore, MXene offers core advantages for designing high-performance, structurally simplified hybrid photocatalytic systems through its intrinsic Fermi level position, high conductivity, and two-dimensional structural characteristics.

MXene, as a pioneering two-dimensional material, has high conductivity like that of precious metals, which is one of its most notable characteristics. This high conductivity stems from its delocalized electron system, enabling it to exhibit excellent performance in electron storage and transport [26,34,41,72]. The unique electronic structure of MXene, characterized by a Fermi level (E_f_) positioned within the conduction band (CB), enables spontaneous electron transfer when interfaced with semiconductors. When MXene’s Ef lies below that of a coupled semiconductor, photoexcited electrons migrate from the semiconductor to MXene, establishing an intrinsic electric field at the interface. This field simultaneously enhances charge separation efficiency and enables directional electron transport across extended spatial domains [75]. The interfacial energy alignment creates a space-charge layer near the semiconductor surface, inducing upward band bending that forms a Schottky junction. This energy barrier effectively suppresses charge recombination by preventing electron backflow into the semiconductor [76]. This highly efficient charge management mechanism has been strongly validated in experiments. Ag_3_PO_4_/Ti_3_C_2_ Schottky photocatalysts prepared using electrostatic self-assembly technology exhibit excellent degradation performance against antibiotics, persistent organic pollutants, and dyes under visible light. Notably, these MXene-based hybrids address the inherent photocorrosion limitations of pristine Ag_3_PO_4_ while outperforming both pure Ag_3_PO_4_ and Ag_3_PO_4_/RGO composites in photocatalytic activity and operational stability [71]. Band engineering through heterojunction design has proven crucial for optimizing charge transfer pathways in photocatalytic systems. Among various configurations, Z-scheme heterojunctions stand out as particularly efficient architectures for directional charge migration while preserving strong redox potentials.

The formation of Z-scheme heterojunctions necessitates stringent criteria, including interleaved band alignment [77], substantial work function disparity [78], and robust interfacial coupling forces [79]. Notably, MXene inherently fulfills these prerequisites for constructing Z-scheme heterojunctions with diverse semiconductors, owing to its tunable electronic structure and surface reactivity (Figure 6c) [80]. Beyond conventional heterojunction engineering in single-component photocatalysts, MXene quantum dots (MQDs) have demonstrated exceptional versatility in modulating interfacial architectures for Z-scheme configurations within multicomposite photocatalytic systems. Mimicking the electron transport chain in natural photosynthesis, Z-scheme heterojunctions enable asymmetric coupling of two semiconductors’ band structures to establish a staggered charge transfer pathway. This architecture drives photogenerated electrons from the conduction band (E_CB_) of Semiconductor A to the valence band (E_VB_) of Semiconductor B, achieving spatial separation of electron-hole pairs. The resultant charge redistribution concentrates holes in semiconductors with high oxidation potentials (e.g., TiO_2_) while accumulating electrons in materials with strong reduction potentials (e.g., g-C_3_N_4_). This synergistic configuration simultaneously preserves the redox capabilities of both components and amplifies photocatalytic driving forces. Through calcination processes, a novel dual Z-scheme heterojunction composed of graphitic carbon nitride/Ti_3_C_2_ MXene/black phosphorus (CN/MX/BP, CXB) was synthesized. This system achieved exceptional photocatalytic degradation efficiency (>99%) for ciprofloxacin (CIP) within 60 min under visible light irradiation (λ > 420 nm) [81]. Similarly, a meticulously engineered In_2_S_3_/Ti_3_C_2_ MXene quantum dot/SmFeO_3_ Z-scheme heterojunction demonstrated 98% sulfamethoxazole degradation efficiency within 120 min under visible light exposure [82]. The enhancement of photocatalytic performance is due to the role of MXene as a metallic conductive electron mediator, which enables the directional transfer of charges. Meanwhile, the surface polarization effect effectively suppresses the recombination of electrons and holes, allowing the optimized interfacial structure to promote the occurrence of multistep redox reactions.

In Z-type heterojunctions, MXene’s unique electronic structure enables a dual key role as an electron acceptor and a hole transport mediator, thereby strengthening bidirectional charge management (Figure 6b). Specifically, the Fermi-level/work-function of Ti_3_C_2_T_x_ is positioned to accept photogenerated electrons from many n-type semiconductors, facilitating electron trapping at the MXene interface and suppressing recombination [83,84]. MXene’s high electrical conductivity promotes rapid interfacial electron extraction from Semiconductor A (e.g., TiO_2_), while its surface terminations (–OH/–O/–F) provide favorable interfacial interactions—including hydrogen bonding—with the valence band of Semiconductor B (e.g., g-C_3_N_4_) (Table 3), enabling directional hole transport within Z-scheme assemblies [85]. In additional, Ti_3_C_2_T_x_ exhibits broadband plasmonic/photothermal response extending from the visible to the near-infrared (≈400–2500 nm), which enhances local electromagnetic fields at MXene–semiconductor interfaces and accelerates carrier migration to reactive sites [86]. Collectively, these effects underpin the “electron-sink” function of MXene in Z-scheme systems and account for the consistently improved photocatalytic performance reported for MXene-coupled heterojunctions in pollutant degradation.

The core strategy for achieving the above performance optimization lies in the construction of photocatalyst composite materials. This strategy aims to improve overall performance through structural optimization, enhanced material/energy transfer, and precise band structure modulation. The core principle involves establishing point-to-surface or surface-to-surface interfacial contacts between materials, where the interfacial characteristics crucially determine the photocatalytic performance beyond individual component properties. These interfaces facilitate synergistic interactions through ① tailored electron transport mechanisms, ② reduced electron-hole recombination rates, and ③ enhanced chemical stability via functional group mediation. As demonstrated in representative systems, Ti mesh-supported flexible g-C_3_N_4_/Ti_3_C_2_/TiO_2_ nanotube arrays exhibited 85.12% degradation of 10 mg/L tetracycline hydrochloride within 180 min, outperforming conventional g-C_3_N_4_/TiO_2_ composites (36% degradation for 20 mg/L under identical conditions) [89]. This enhancement arises from two synergistic mechanisms: ① Formation of Z-scheme heterojunctions between g-C_3_N_4_ and Ti_3_C_2_ and ② Strong interfacial coupling between Ti substrate and composite materials.

The MXene surface is rich in functional groups such as -OH, -O, and -F, providing an ideal basis for chemical modification and light-active semiconductor loading as a two-dimensional platform. This enables semiconductor photocatalysts to self-assemble in situ on MXene to form composite materials while also facilitating the loading of metal oxides (such as Fe_3_O_4_ [90], Ag_3_PO_4_ [91]) to construct high-performance composite photocatalytic systems. Room-temperature synthesized TCT (TiO_2_ NPs/C-doped amorphous TiO_x_ homojunction with residual Ti_3_C_2_T_x_ MXene cocatalyst) achieved 91.5% tetracycline degradation within 100 min, maintaining 86.9% efficiency after six cycles. The Type-II heterojunction configuration promotes directional charge separation [92]. Magnetically responsive MXene hybrids (Fe_2_O_3_ nanoparticles anchored on Ti_3_C_2_ surface) designed by combining heat treatment and hydrothermal method. This composite demonstrates enhanced tetracycline removal efficiency (92.4% within 60 min) and rapid magnetic separation capability (saturation magnetization: 15.2 emu·g^−1^), while the Fe_2_O_3_ incorporation effectively mitigates the inherent stability limitations of pristine MXene under oxidative conditions [90]. This method of strategically combining MXene with structurally compatible materials (such as semiconductors and metal oxides) successfully addresses the key shortcomings of single component photocatalysts, namely rapid charge recombination and insufficient active sites. Furthermore, MXene’s transition metal matrix enables in situ growth of semiconductor hybrids (e.g., TiO_2_-C [89]) through its role as both a structural template and electron mediator. A summary of the photocatalytic-promoting functions of MXene is presented in Table 4.

### 3.3. The Chemical Structure of MXene Strengthens the Stability of the Material

#### 3.3.1. Regulation Strategies for Strong Metal–Support Interactions in MXene Photocatalytic Systems

The emerging strong metal–support interaction (SMSI) offers a transformative paradigm for designing MXene-based photocatalytic systems. Owing to their lamellar architecture, tunable surface terminations, and metallic-like conductivity, MXenes provide robust platforms for SMSI-type electronic coupling and interfacial configuration control, enabling advances in carrier separation, active-site stabilization, and photo-corrosion resistance [94].

Surface terminations (–O/–F/–OH) participate in p-d orbital hybridization with supported metals and semiconductors, acting as interfacial electron buffers and modifying band alignment. In Au/Ti_3_C_2_T_x_ systems, experiments and calculations show substantial charge redistribution at the interface and improved interfacial transport, consistent with an SMSI-like electronic contact that favors directional charge flow under illumination [95]. Beyond static interfacial states, photo- or thermo-induced SMSI is known to restructure metal/support contacts and form ultrathin encapsulation layers on classical oxide supports; analogous interfacial reorganization pathways (e.g., Ti–O–M linkages, defect-assisted bonding) are increasingly explored on 2D supports and MXenes to stabilize active phases during photocatalysis [96]. For MXene/transition-metal dichalcogenide (TMD) junctions (e.g., V_2_C//MoS_2_), interface engineering and covalent/defect-anchored bonding have been shown to strengthen electronic coupling, tune the local work function/d-band center, and optimize adsorption energetics of intermediates—collectively promoting bidirectional charge transfer and durability [97]. In parallel, MXenes exhibit broadband optical/photothermal responses (visible-to-NIR) that intensify local electromagnetic fields and enhance multi-field energy coupling at the metal/MXene or semiconductor/MXene interface, which is beneficial for light harvesting and hot-carrier utilization.

Altogether, these SMSI-mediated strategies—combining termination-guided electronic/geometric effects, adaptive interfacial protection, and broadband light–matter coupling—provide practical design rules for stable, high-efficiency MXene-based photocatalytic systems applicable to solar water splitting, CO_2_ reduction, and pollutant degradation.

#### 3.3.2. Case Studies of Enhanced Stability in MXene Photocatalytic Systems

MXene, with its unique structure rich in transition metals such as titanium, shows great potential as a multifunctional composite catalyst. The surface-exposed Ti sites on MXene exhibit significantly enhanced redox reactivity compared to conventional carbon materials, facilitating in situ redox cycling of metal oxide catalysts and improving photocatalytic stability. Theoretical studies confirm that these Ti sites accelerate electron transfer kinetics, with measured rate constants exceeding those of noble metal cocatalysts by 2–3 orders of magnitude. Simultaneously, the abundant surface Ti and transition metal sites stabilize metal oxides through dual mechanisms: (1) enhancing synergistic catalytic pathways via optimized charge transfer, and (2) suppressing metal leaching by passivating lattice dissolution channels.

MXene’s excellent interface control capability is the key to its performance improvement. The hydrophilic surface functional groups (-OH, -O) of Ti_3_C_2_ enable intimate interfacial contact with semiconductors like Ag_3_PO_4_. This interaction, combined with MXene’s redox-active Ti sites, mitigates Ag+ reduction by photoelectrons, reducing photocorrosion by 78% compared to bare Ag_3_PO_4_ [41]. In Cu_2_O/Cu@MXene nanocomposite, MXene substrate simultaneously enhances tetracycline (TC) adsorption and interfacial electron transfer, achieve 99.14% tetracycline removal within 30 min (pseudo-first-order rate constant: 0.1505 min^−1^), representing a 3.2 times enhancement over standalone Cu_2_O/Cu, and the degradation rate remains above 82% after five cycles [98].

Photocorrosion resistance remains a critical challenge for chalcogenide semiconductors (e.g., CdS, ZnS [99]), where surface sulfide ions (S^2−^) oxidize to elemental sulfur or sulfates during photocatalysis, leading to structural degradation. The 1D CdS/2D MXene heterojunction demonstrates exceptional stability, maintaining consistent hydrogen evolution rates over 15 h of continuous operation [100], in stark contrast to pristine CdS, which exhibits an activity loss within 8 h [101]. This highlights MXene’s dual role as both a charge-transfer mediator and protective cocatalyst to suppress photocorrosion. Further stability enhancements are achieved through polymer-MXene nanocomposite engineering. Polyvinylpyrrolidone (PVP)-encapsulated MXene cast onto polyethylene terephthalate (PET) substrates forms PMP photoresponsive films for tetracycline degradation. MXene incorporation modulates interlayer spacing (increasing from 0.98 nm to 1.24 nm) and composite surface roughness (Ra = 12.3 nm vs. 8.7 nm for pure PET), optimizing active site distribution and reducing photocatalytic deactivation risks [15]. More importantly, MXene’s large specific surface area makes it an ideal catalyst growth substrate, effectively preventing active component aggregation and inhibiting deactivation, and its functionality is superior to that of precious metals that require high specific surface area carriers [102].

MXene quantum dots (MQDs) offer unique advantages for constructing efficient photocatalytic systems due to their ultra-high specific surface area (approximately 300 m^2^/g), strong quantum confinement effect, and excellent metallic conductivity. MQD can act as intermediate electron pathways to form Z-scheme heterojunctions, thereby enhancing photocatalytic reactions. MQDs with lateral dimensions below 20 nm circumvent the shielding effects commonly observed in noble metal nanoparticles [26], while the Schottky junctions formed between MQDs and photocatalysts enhance carrier mobility and interfacial coupling strength [71,100]. Ti_3_C_2_T_x_ MQDs exhibit exceptional antibacterial properties under low-concentration, dark conditions, maintaining efficacy after 4 h of continuous mechanical agitation [103]. Integrating MQDs into photocatalytic systems enables innovative exploration of their bacteriostatic mechanisms. Leveraging strong quantum confinement, metallic conductivity, anisotropic charge transport, and high surface area, MQDs serve as rapid electron highways in Z-scheme heterojunctions, effectively suppressing bacterial adhesion and biofilm formation. This reduces corrosion and fouling from microbial metabolites, extending photocatalyst lifespan. MQDs excel in generating reactive oxygen species (ROS) under UV irradiation, which are crucial for the efficient degradation of antibiotics. For instance, a g-C_3_N_4_/BiOBr/MQD composite synthesized via solvothermal methods combines heterojunctions, oxygen vacancies (OVs), and MQD-enhanced charge transfer. OVs promote O_2_ chemisorption, while MQDs accelerate electron transport and selectively generate ^1^O_2_, achieving 99% tetracycline hydrochloride degradation within 30 min under visible light. Long-term stability tests reveal >90% efficiency retention after 8 cycles (40 min each) [104]. By exploiting MXene’s structural, chemical, and reactive versatility, this approach resolves persistent challenges like photocorrosion in conventional semiconductors, enhancing both stability and operational durability.

### 3.4. Photocatalytic Composites for Co-Catalytic Modification with MXene Co-Catalytic

To further explore MXene as a co-catalyst to enhance photocatalytic performance and accelerate the rate of photocatalytic reactions, the photothermal effect, localized surface plasmon resonance (LSPR) effect generated under the regulation of MXene co-catalysis, as well as the synergistic effects with other modification methods were investigated, to clarify the versatility and practicality of MXene as a co-catalyst.

#### Innovative Mechanisms for Plasma-Induced Chemical Reactions

MXene exhibits excellent interface compatibility in photocatalytic systems, with advantages in terms of broad spectral response and low defect interfaces (Table 5). Its localized surface plasmon resonance (LSPR) effect enables broadband infrared absorption extending beyond 800 nm, effectively addressing the narrow optical response range characteristic of conventional semiconductors [49]. The atomically smooth surface morphology (root-mean-square roughness < 0.2 nm) and inherent two-dimensional architecture promote robust van der Waals interactions (binding energy ≈ 0.03 eV/atom) with semiconductor substrates, resulting in a 62% reduction in interfacial defect density compared to conventional heterojunctions.

Metal nanostructures generate a plasmonic effect upon photoexcitation and trigger chemical reactions through two core mechanisms. When the energy of the incident light (hv) exceeds the Fermi level (Ef) of the metal, non-equilibrium state hot electrons are generated. Subsequently, the energy is transferred to the lattice via electron–phonon coupling within a picosecond time scale. At the same time, the vibrational energy of the lattice is transferred to the surface molecules through interfacial vibrational coupling. These two mechanisms (hot electron transfer and phonon-mediated heat transfer) work synergistically to enhance the efficiency of MXene-based photocatalytic systems.

MXene, as a new class of two-dimensional transition metal carbide, has entered the advanced plasmonic material system due to its unique electronic structure characteristics. Compared with traditional materials, it exhibits three breakthrough characteristics in the field of light-matter interaction.

First, it shows a broadband plasmonic response. A significant surface plasmon resonance effect is presented in the visible to near-infrared wavelength range (400–2000 nm), and its resonance frequency can be tuned by the number of layers (a single-layer red-shift is about 15%). Compared with noble metals (Au/Ag only cover the visible light region), the plasmonic absorption bandwidth of MXene can be extended by 3–5 times, providing the possibility for multi-wavelength light energy capture. Secondly, the surface termination group engineering can induce significant surface-shell synergistic effects. Under surface engineering modification, terminating groups such as -O and -F regulate the position of the Fermi level through p-d orbital hybridization (the regulation range can reach 0.8 eV). More importantly, the atomic-level flatness of MXene’s surface (roughness < 0.2 nm) and the weak interlayer van der Waals interactions (binding energy ~0.03 eV/atom) work together to give it an exceptional ability to precisely control the direction of molecular adsorption. In addition, it has other advantages compared with traditional photocatalysts in Table 6.

Ti_3_C_2_T_x_ MXene exhibits exceptional photothermal conversion efficiency, enabling direct solar-to-thermal energy transduction within catalytic systems. The localized surface plasmon resonance (LSPR) effect amplifies interfacial electromagnetic fields (field intensity enhancement: 10^2^–10^3^), thereby reducing activation energy barriers (ΔE ≈ 0.35 eV) and enhancing both thermodynamic feasibility and kinetic rates of photocatalytic degradation [30,111]. Thickness-dependent studies reveal that Ti_3_C_2_T_x_ nanosheets with sub-10 nm dimensions achieve 1.8 times stronger LSPR fields than bulk counterparts, positioning MXene as a cost-effective alternative to noble metals for plasmonic applications [112]. Under illumination, plasmonic Ti_3_C_2_T_x_ generates high-energy hot holes that directly oxidize antibiotics and organic pollutants into low-molecular-weight intermediates. Concurrently, delayed electron-hole recombination enables hot electrons to migrate toward adsorbed O_2_, generating reactive superoxide radicals (O^2−^) for pollutant mineralization [112,113]. In the photothermal effect, it has been found that the construction of heterojunctions increases the electron cloud density of Ti_3_C_2_T_x_. Meanwhile, it also enhances the number of hot electrons and the intensity of the local electromagnetic field generated by the localized surface plasmon resonance (LSPR) [114]. During the photocatalytic reaction process, the separation of photogenerated electron-hole pairs and the migration of photogenerated electrons occur under photoexcitation. At the same time, the thermal effect also provides a certain amount of energy for the photoelectrons, which serves as one of the driving forces for their separation and migration.

Surface defects commonly found in solid materials, particularly surface oxygen vacancies (OVs), play a central role in photocatalysis. OVs, as the imperfect lattices with rearranged electron distributions left by the loss of oxygen atoms in the semiconductor crystal structure, are the most common anion vacancies [115]. They are conducive to the formation of active sites that are crucial for the catalytic process. The escape of oxygen atoms from the lattice sites of semiconductors leads to the redistribution of electrons. By promoting the exposure of active sites and narrowing the bandgap, it improves the physical and chemical properties of the material, thus optimizing the kinetics of various reactions, and has attracted extensive attention especially in the field of photocatalysis [116]. Integrating the main catalyst with MXene nanomaterials forms a multicomponent heterojunction. The lattice mismatch and lattice distortion caused by the different crystal structures of each component induce the generation of interfacial oxygen vacancies (OVs), as shown in Table 7. The formation of oxygen vacancies captures many photogenerated electrons produced under photoexcitation and inhibits the recombination of photogenerated electron-hole pairs. The electrons occupying the O 2p orbitals can significantly activate the oxygen-deficient surface as an electron-rich center, thereby providing more oxygen adsorption sites, reducing the bandgap of the semiconductor, promoting the separation of electron-hole pairs, and exerting an enrichment effect on electrons and pollutants, thus enhancing the reaction ability under visible light.

Leveraging MXene’s intrinsic properties (e.g., tunable interlayer spacing, metallic conductivity) and co-catalytic optimization mechanisms, MXene composites with metal oxides [123], metal sulfides, graphitic carbon nitride (g-C3N4) [124], and oxyhalides [125] have emerged as robust platforms for antibiotic degradation. These composite systems effectively address two key challenges: (1) enhancing operational stability and (2) amplifying photoactivity via extended visible-light absorption and charge separation efficiency.

Developed by the Hefei Institutes of Physical Science, this architecture exploits MXene’s biocompatibility and interlayer spacing (0.98–1.25 nm adjustable) to in situ grow Cu_2_O/Cu nanoparticles. The system achieves 92% tetracycline removal within 60 min by synergizing adsorption-enriched tetracycline at MXene interfaces with accelerated electron transfer to Cu sites (charge transfer resistance reduced by 68%) [126]. Integrating MXene quantum dots (MQDs) with phosphorus-doped graphitic carbon nitride (g-C_3_N_4_) creates a 0D-2D heterostructure that enhances photocatalytic efficiency through dual mechanisms: (1) increased specific surface area and (2) suppressed charge recombination. This architecture demonstrates exceptional tetracycline hydrochloride (TC) degradation (88.40% in 60 min) and Cr_(VI)_ reduction efficiency (94.2% in 45 min) under visible light.

The aerosol-assisted self-assembled g-C_3_N_4_/MXene/Ag_3_PO_4_ (PCN/M/AP) S-scheme heterojunction exhibits MXene-dependent activity enhancement. Increasing MXene content from 3 wt% to 7 wt% elevates TC degradation rates from 53.79% to 88.40% [91]. Under visible light irradiation, the rate constant value of PCN/M/AP is approximately 4.3 times higher than that of the pristine g-C_3_N_4_. This is attributed to the formation of a MXene-mediated Z-scheme heterojunction, which promotes the separation and transfer of photogenerated electron-hole pairs. In addition, after 5 consecutive cycles, the photocatalytic performance of PCN/M/AP for tetracycline (TC) still remains at 83.80% [127]. In MXene-based composites, g-C_3_N_4_ is widely used mainly due to its unique advantages. On one hand, it has advantages such as suitable bandgap energy, high chemical stability, a unique layered structure, and non-toxicity [80]. Compared with other metal-containing semiconductors, such as metal sulfides and metal oxides, metal-free g-C_3_N_4_ will generate a strong C-N conjugate structure after high-temperature calcination, ensuring excellent stability and resulting in the high photocatalytic ability of g-C_3_N_4_. On the other hand, the conjugate structure derived from triazine units and the two-dimensional structure caused by the weak van der Waals force between adjacent layers both create abundant active sites for contact with target pollutants. However, the visible light reaction rate of g-C_3_N_4_ is relatively low because its bandgap (Eg) is only 2.7 eV, and it only absorbs light with a wavelength less than 470 nm. At the same time, the recombination of electrons and holes easily occurs on g-C_3_N_4_, leading to a significant decrease in photocatalytic efficiency [128,129,130]. MXene is the key component that overcomes these limitations. As an efficient intermediate, MXene can not only achieve the rapid transfer of photogenerated electrons to enhance the overall photocatalytic activity but also construct more mature and effective Z-scheme heterojunctions to strongly suppress carrier recombination.

## 4. Dynamic Regulation of MXene Coordination Structure and Photocatalytic Environmental Adaptability

### 4.1. Photocatalytic Anti-Interference Mechanism in Complex Water Quality Environment

MXene exhibits multifunctional roles in photocatalytic antibiotic degradation, primarily mediated by its tunable surface/electronic properties governed by microstructure and surface chemistry. To comprehensively evaluate its synergistic catalytic performance, it is necessary to systematically analyse the effects of external environmental factors (pH value, coexisting ions and dissolved organic matter) on the system’s performance and stability.

The initial wastewater pH critically modulates photocatalytic activity through two mechanisms: (1) altering adsorbent surface charge via the point of zero charge and (2) inducing pH-selective antibiotic interactions. Below pH 6.85 (acidic to near-neutral conditions), electrostatic attraction between the positively charged catalyst surface and tetracycline hydrochloride (TCH, pKa ≈ 3.3) enhances adsorption capacity (Qe: 48.7 → 92.4 mg·g^−1^) [131]. Conversely, at pH > 6.85, reduced surface charge density weakens catalyst-antibiotic affinity, decreasing degradation efficiency (89% → 63% over pH 4–9). The acid dissociation constant (pKa) quantifies the pH-dependent ionization behavior of weakly acidic/basic antibiotics, defining the pH at which 50% dissociation occurs. Antibiotic-specific pKa values (Table 8) govern their ionization states: below pKa, neutral molecular forms dominate (e.g., sulfamethoxazole, pKa = 1.7/5.6), while ionized species prevail at pH > pKa (e.g., tetracycline, pKa = 3.3/7.7/9.7) [132]. This ionization dichotomy critically modulates adsorption selectivity on photocatalytic polymer surfaces. Cationic antibiotics (e.g., ciprofloxacin, pKa = 6.1/8.7) exhibit 3.2 times higher adsorption capacity (Q_max_ = 128 mg·g^−1^) on negatively charged MXene-PLA composites at pH 7.4 via electrostatic attraction. Anionic antibiotics (e.g., sulfadiazine, pKa = 2.0/6.5) preferentially bind to protonated amine-functionalized polymers (Q_max_ = 98 mg·g^−1^) at pH 4.2. The differential adsorption arises from pH-tunable surface charges (zeta potential range: −35 mV to +28 mV) and chemical affinity mismatches (hydrophobic/hydrophilic balance ΔH ≈ 12–18 kJ·mol^−1^). Molecular dynamics simulations confirm that ionized antibiotics exhibit 1.8–2.5× stronger binding energies (E_bind_ = −45 to −68 kJ·mol^−1^) compared to neutral forms due to enhanced electrostatic/hydrogen-bond interactions [133]. The degradation efficiency of antibiotics is governed by both molecular ionization states and catalyst structural stability. See Table 8 for details. Amphoteric antibiotics like tetracycline and amphotericin B exhibit pH-dependent charge switching, modulating their adsorption affinity to photocatalysts [134].

The presence of inorganic anions (e.g., HCO_3_^−^, Cl^−^) in real aqueous systems introduces competitive interfacial dynamics that govern antibiotic removal efficiency. HCO_3_^−^ enhances tetracycline-class antibiotic degradation (e.g., oxytetracycline and chlortetracycline) through •OH radical amplification, whereas Cl^−^ exhibits dual inhibitory effects: (1) •OH scavenging and (2) catalyst surface passivation, collectively suppressing oxytetracycline and chlortetracycline degradation by 14.8% at 100 mmol L^−1^ with concentration-dependent inhibition trends. These anions further modulate antibiotic mobility by altering photocatalyst surface charge, promoting nanoparticle aggregation and reducing environmental transport efficiency. Such ion-specific interfacial engineering highlights the need for tailored photocatalyst design to mitigate competitive anion effects in complex wastewater matrices.

Humic acid is the main component of total organic carbon (TOC) in water (about 50%) and mainly acts as a free radical scavenger in photocatalytic systems. At 100 mg L^−1^ humic acid, TC-HCl removal decreases marginally from 99% to 94% due to competitive •OH consumption (quenching efficiency: 73.5%). Notably, humic acid preferentially adsorbs on TiO_2_ surfaces (Qmax = 185 mg g^−1^), increasing isoelectric points (IEP: 5.8 enhanced 7.2) and promoting nanoparticle agglomeration (hydrodynamic diameter: 45 enhanced 210 nm) [104].

### 4.2. Dynamic Optimization Strategy of the Coordination Structure of MXene

MXene-based composites demonstrate excellent photocatalytic performance and stability through sophisticated interface engineering. In Pd/MXene/MIL-101(Fe) heterojunction, synergistic interfacial effects significantly enhanced visible light absorption (>500 nm) and charge separation efficiency, achieving 98% efficient degradation of ofloxacin [137]. MXene’s surface functional groups (-OH, -F) optimize electronic coupling with MIL-101(Fe), while Pd modulates OFL intermediate binding energies, enabling pH-selective adsorption and radical-dominated degradation. Pd/MXOF maintains >90% efficiency across pH 4–10 due to MXene’s pH-responsive surface charge and MIL-101(Fe)’s adsorption enrichment.

Using two-dimensional Ti_3_C_2_ MXene as the matrix material, in the presence of a crystal plane controlling agent, the (001)-facet-exposed TiO_2_/Ti_3_C_2_ MXene photocatalyst was prepared by a hydrothermal reaction method at 160 °C [59]. The formation of the heterojunction on the TiO_2_ surface, the Schottky junction at the interface between (001)-facet TiO_2_ and Ti_3_C_2_ MXene, and the LSPR effect of Ti_3_C_2_ MXene on high-energy hot electrons, hot holes, and the local magnetic field synergistically promoted the spatial separation of photogenerated electrons and holes. The carrier complexation was effectively inhibited, and the near-infrared light and full-spectrum catalytic activities of the (001) TiO_2_/Ti_3_C_2_ photocatalyst were improved [59].

Through the interface reconstruction between carbon dots and MXene-Ti_3_C_2_/CeO_2_, a three-phase micro-heterojunction was formed, enhancing the local active site density. The optimized catalyst increased the ammonia production rate by 8 times under visible light, which is attributed to the dynamic matching of oxygen vacancies and surface charges [138]. When the concentration of humic acid reached 100 mg L^−1^, the photocatalytic efficiency only decreased by 5%, indicating the anti-inhibition ability of MXene-based materials against natural organic matter.

## 5. Research Prospects and Outlook

### 5.1. Opportunities and Challenges

Although MXene shows unique advantages in the field of photocatalysis, its practical application still faces the following core challenges:

① The preparation of MXene mainly relies on the etching of MAX-phase precursors with hydrofluoric acid (HF) or fluorine salts (such as LiF + HCl). This method has problems such as complex processes, low yields (<50%), and toxic by-products (such as AlF_3_). In addition, the lateral size of MXene nanosheets is usually 1–5 μm, and it is difficult to achieve large-area (>10 cm^2^) uniform film formation through existing technologies (such as liquid-phase exfoliation). Studies have shown that the defect density of MXene films increases exponentially with the increase in area, resulting in a significant decrease in photocatalytic activity (for example, the hydrogen production efficiency of a 10-cm^2^ film is only 30% of that of small-piece samples).

② The rich active functional groups (-OH, -O) on the surface of MXene endow it with excellent adsorption properties but also make it prone to irreversible oxidative degradation in humid, high-temperature, or oxidative environments. For example, after Ti_3_C_2_T_x_ is exposed to air for 72 h, TiO_2_ nanoparticles are formed on the surface due to oxidation, the electrical conductivity decreases by >80%, and the photocatalytic activity is lost by >60%. In wastewater containing Cl^−^ or SO_4_^2−^, ion intercalation easily occurs between the layers of MXene, accelerating the structural collapse.

③ The wide-spectrum absorption (ultraviolet-visible-near-infrared) characteristics of MXene are often accompanied by a high carrier recombination rate (lifetime <10 ps), and its metallic surface leads to the rapid recombination of photogenerated electron-hole pairs. Although the heterojunction design can partially alleviate this problem, interface defects (such as dangling bonds, lattice mismatch) still limit the improvement of quantum efficiency (usually <20%).

In response to the above challenges, the following breakthrough strategies have been proposed in recent studies:

① Coating an inert protective layer (such as SiO_2_, Al_2_O_3_) on the surface of MXene by atomic layer deposition (ALD) or sol–gel method can significantly improve its environmental stability. For example, SiO_2_-coated Ti_3_C_2_T_x_ (SiO_2_@Ti_3_C_2_T_x_) reduces the degree of oxidation by 90% after being stored at 85% humidity for 30 days, and the efficiency of photocatalytic degradation of tetracycline remains 85% of the initial value. The mechanism lies in that the SiO_2_ layer (with a thickness of 2–5 nm) blocks the penetration of H_2_O and O_2_ while retaining the surface-active sites of MXene.

② Introducing sulfur vacancies (S-vacancy) or nitrogen doping (N-doping) into MXene through plasma treatment or chemical doping can optimize its energy band structure and carrier dynamics. For example, the hydrogen production rate of sulfur-vacancy-modified Mo_2_CT_x_ (Mo_2_CT_x_-Sv) under visible light reaches 8.2 mmol·g^−1^·h^−1^, which is 3.5 times that of the original sample. Density functional theory (DFT) calculations show that sulfur vacancies act as electron traps, extending the carrier lifetime to 50 ps, and improving catalytic selectivity by reducing the adsorption energy of H intermediates (ΔGH decreases from 0.45 eV to 0.18 eV).

③ Developing fluorine-free etchants (such as the NaOH melting method) and continuous roll-to-roll film-forming technology can consider both environmental protection and large-scale production. For example, the yield of Nb_2_CT_x_ synthesized by the KOH-assisted hydrothermal method is increased to 78%, and the emission of toxic fluorides is avoided. In addition, the bionic dynamic interface design (such as MXene/hydrogel composites) realizes pH-temperature-responsive structural reorganization through the self-repair ability of the hydrogen-bond network, maintaining the stability of catalytic activity under extreme conditions (pH = 2–12).

### 5.2. Take a Reasonable Look at the Future of MXene

MXene’s 2D architecture offers unparalleled opportunities for composite photocatalyst engineering through three synergistic pathways: (1) As an oxidative growth platform, controlled partial oxidation of Ti_3_C_2_ enables in situ TiO_2_ heterojunction formation, though precise regulation of oxidation kinetics and dopant distribution remains critical to optimize optical band structures and interfacial electron transfer rates [139]. (2) Terminal group (-OH/-O/-F) modulation via selective etching or post-synthetic functionalization dynamically tunes MXene’s work function, enabling adaptive Schottky barrier engineering with semiconductors like g-C_3_N_4_. (3) Environment-responsive surface restructuring—protonation in acidic media (pH < 4) or oxidative radical attack during photocatalysis—alters Ti redox states and photothermal conversion efficiency. While DFT simulations reveal substrate-specific electronic state modulation, practical deployment requires overcoming HF-dependent synthesis limitations (toxic AlF_3_ byproducts, <50% yield) through eco-friendly molten-salt alternatives (KOH/NaOH etching, 75–85% purity). Multiscale modeling spanning electronic interactions to reactor-level photothermal-fluidic coupling is essential to decode MXene’s adaptive functionality in complex aqueous matrices and accelerate solar-driven water remediation.

## 6. Conclusions

MXenes, a unique class of two-dimensional transition metal carbides, nitrides, and carbonitrides, have emerged as highly promising photocatalytic materials owing to their large specific surface area, tunable surface terminations, and metallic-like conductivity. Advances in etching technologies and post-synthetic modification have enabled precise regulation of MXenes’ morphology, interlayer spacing, and surface chemistry, which in turn determine their electronic structures, charge-transfer dynamics, and catalytic efficiency.

Functionally, MXenes can serve both as electron acceptors and as efficient charge-transport mediators, thereby effectively suppressing electron–hole recombination and enhancing the performance of semiconductor-based heterojunctions. These distinctive properties endow MXenes with broad applicability: they can act as cocatalytic platforms for pollutant degradation and antibiotic removal, as structural templates for hierarchical architectures, and as core components in Z-scheme photocatalytic systems. Of particular importance in polymer-based photocatalysis, MXenes’ adaptable interfacial characteristics—governed by protonation/deprotonation kinetics, electrochemical polarization effects, and environmental conditions—provide a new pathway for constructing responsive and programmable catalytic processes. By integrating MXenes into polymer matrices, one can achieve synergistic effects that enhance light harvesting, charge separation, and catalytic durability, thus enabling multifunctional and sustainable photocatalytic platforms.

Despite these advances, several challenges remain. MXenes are prone to oxidative degradation, face difficulties in large-scale synthesis, and often exhibit limited stability under complex aqueous conditions. To overcome these limitations, innovative strategies are required, such as applying protective surface coatings, engineering defects/dopants, developing environmentally friendly fluorine-free synthesis routes, and learning from biological systems to design dynamic interfaces. Meanwhile, the integration of density functional theory (DFT), multiscale simulations, and machine learning provides a powerful bridge between theoretical predictions and experimental outcomes, accelerating the rational design and application of MXene-based systems.

Looking ahead, MXene–polymer composites hold great potential to advance from laboratory studies to scalable practical applications. Their unique adaptability, arising from the interplay of electronic structures and surface chemistry, positions MXenes as transformative building blocks for next-generation high-performance and sustainable photocatalytic systems in environmental remediation, solar-driven energy conversion, and advanced separation technologies.

## Figures and Tables

**Table 1:** Comparison of the degradation performance of different photocatalytic composite materials on tetracycline.

Photocatalysts	Tetracycline (mg L^−1^)	Reaction Time	The Dosage of Photocatalyst (mg)	Removal Rate (%)	Rate Constant k (min^−1^)	Quantum Efficiency (%)	Reference
D–OM–ZIF-8/ZnO	1000	Irradiate with ultraviolet-visible light for 60 min.	50	88.5 90.5	0.048	2.9	[9]
CF/ZnO/Ag_2_O	20	Irradiate with a 500 W xenon lamp for 30 min.	50	94.5	0.08368	3.8	[10]
BiOI/Brookite TiO_2_	20	Irradiate with visible light for 110 min.	30	82.0	0.063	4.8	[11]
Ag_2_CO_3_/ZIF-8/CF	10	Irradiate with visible light for 30 min.	30	92.0	0.076	5.1	[12]
CeO_2_/BiYO_3_	30	Irradiate with visible light for 60 min.	50	90.0	0.085	5.5	[13]
Ti_3_C_2_T_X_/Cu_2_O	30	Irradiate with visible light for 40 min.	30	97.6	0.091	6.0	[14]
PVP-MXene-PET	0.1	visible light for 50	1	83.01%	-	-	[15]
MXene-PVA-TiO_2_	50 mL, 2 mg/L (MB)	a 300 W Xe lamp for 9 h	-	95.2%	-	-	[16]
PVP/PEO/MXene Nanocomposite	2 ppm (MB)	500–800 nm for 120 min	5 wt% of MXene	61.6%	0.08	-	[17]

**Table 2:** MXene features optimized photocatalysis for other semiconductors [41,60,61].

Advantage	The Deficiencies Existing in Other Semiconductor Photocatalysis	The Mechanism of the Enhanced Photocatalytic Effect of MXene
Abundant catalytic active sites	The catalytic sites are occupied due to the influence of environmental factors.	Construct composites by loading single atoms, clusters and nanoparticles on the surface to provide multiple sites.
Abundant surface functional groups	Poor stability	Bond with the main catalyst in the form of hydrogen bonding chemical bonds to improve stability.
Transition metals possess relatively high redox ability.	The phenomenon of photocorrosion	The high redox activity of transition metals inhibits the photocorrosion phenomenon.
Broad spectral response range	A relatively low light response range	Composites formed by mechanical mixing, self-assembly and in situ oxidation have a relatively high light response range
Lower Fermi level compared with common semiconductors	High recombination rate of photogenerated electrons and holes	Construct structures such as Schottky heterojunctions to inhibit the recombination of photogenerated electrons and holes.
High electrical conductivity	The transportation efficiency of photogenerated electrons is not high.	MXene acts as an electron acceptor to improve electron transfer efficiency.

**Table 3:** Band structure matching requirements [60,87,88].

Parameter	Semiconductor A (Reduced Prototype)	Semiconductor B (Oxidized Type)	MXene Roles
Band Position (E_CB_)	High (e.g., g-C_3_N_4_)	Low (e.g., TiO_2_)	Electron transfer bridges
Valence Band Position (E_VB_)	Lower (e.g., g-C_3_N_4_)	High (e.g., TiO_2_)	Hole transport channels
Differences in work function (ΔΦ)	≥0.5 eV		Drive charge separation

**Table 4:** MXene co-catalytic optimization function [23,41,93].

Function		MXene
Photocatalyst preparation	Growth platform	Surface functional groups such as -OH, -O, -F, etc.
	Semiconductor precursors	Metastable transition metal atoms
Improved photocatalytic activity	Electronic receivers	High electrical conductivity and good band structure
	Active site	Multi-purpose transition metal atoms
	adsorbent	Electrostatic attraction
Enhanced photostability	Avoid photoelectron reduction	Transfer of photogenerated electrons

**Table 5:** Spatiotemporal properties of interfacial energy transfer.

Process	Time Scale	Dominant Mechanism	Effect on Reaction	Reference
Thermal electron generation	<100 fs	Electron excitation	Charge transfer initiates redox	[105]
Electron–phonon relaxation	1–10 ps	Energy localization	The reaction temperature field is regulated	[106]
Interface vibration coupling	10–100 ps	Molecular vibrational mode excitation	Reduces the activation energy of the reaction	[107]

**Table 6:** Comparison of performance with traditional materials.

Parameter	MXene	Precious Metal Nanoparticles	Semiconductor Nanosheets	Reference
Light absorption range	Visible infrared	Visible light	The bandgap is decided	[74]
Carrier mobility	10^3^–10^4^ cm^2^/Vs	Limited by size effect	Usually <100 cm^2^/Vs	[108]
Surface reactivity	Can be chemically modified	It depends on crystal plane exposure	Governed by defective states	[41]
Photothermal conversion efficiency	92% (808 nm)	65–75% (Au)	Affected by light absorption characteristics	[95,109]
Hot carrier lifetime	150–300 fs	10–50 fs	Interaction of interfaces	[110]

**Table 7:** Synergistic Degradation of Antibiotics by MXene Co-catalytic Materials Modified with Oxygen Vacancies and Elemental Doping.

Categories	Photocatalytic Materials	Synthesis Method	Contaminant	Reaction Conditions	Degradation Efficiency	Reference
Introduction of oxygen vacancies	NiFe-LDH/MXene	Hydrothermal method	Norfloxacin (20 mg/L)	300 W Xenon lamps 25 °C ± 1 °C	4 h, 98%	[117]
	BiOBr/MXene/gC_3_N_4_	Electrostatic self-assembly	Tetracycline (20 mg/L)	300 W Xenon lamps 25 °C ± 1 °C	30 min, 99%	[104]
	Bi_2_O_2_CO_3_/Ti_3_C_2_T_x_	Hydrothermal method	Levofloxacin (20 mg/L) Amoxicillin, tetracycline (20 mg/L)	300 W Xenon lamps 25 °C ± 1 °C	80 min, 95.4% 30 min, 90.9% and 82.8%	[116]
Doping of metallic elements	Sm doped g-C_3_N_4_/Ti_3_C_2_MXene	Annealing	Ciprofloxacin (20 mg/L)	300 W Xenon lamps 25 °C ± 1 °C	60 min, 99%	[118]
	Tb^3+^ and Mg^2+^ doped CdAl_2_O_4_@MXene	Co-precipitation Sonication	Aspirin (20 mg/L)	300 W Xenon lamps 25 °C ± 1 °C	135 min, 79.6%	[119]
	Co doped ZnTiO_3_/Ti_3_C_2_T_x_ MXene	Liquid self-assembly	Tetracycline (20 mg/L)	300 W Xenon lamps 25 °C ± 1 °C	90 min, 91.5%	[49]
	Fe doped with magnetism MXene/g-C_3_N_4_	Hydrothermal synthesis	Clindamycin (initial drug concentration 125 mg/L) mix with wastewater	100 mW/cm^2^ of high-voltage lamps 25 °C	120 min, 92%	[120]
Doping of non-metallic elements	N,P-MXene/ZnIn_2_S_4_ Schottky knot	In situ grown	Ciprofloxacin (20 mg/L)	300 W Xenon lamps 25 °C ± 1 °C	120 min 99.3%	[121]
	Homojunctions of C-doped amorphous TiO_x_ derived from TiO_2_ and Ti_3_C_2_T_x_	Hydrogen peroxide oxidation	Tetracycline (30 mg/L)	300 W Xenon lamps 25 °C ± 1 °C	100 min, 91.5%	[92]
	MXene-derived carbon-doped TiO_2_ coupled with porous g-C_3_N_4_	One-step hot calcination method	Ciprofloxacin hydrochloride (20 mg/L)	300 W Xenon lamp 420 nm UV filter 25 °C ± 1 °C	50 min, 88.14%	[122]

**Table 8:** Antibiotic classification, structure and pKa value [135,136].

Major Classes of Antibiotics	Secondary Classification	Category	pKa	Structure
β-lactam antibiotics	Penicillins	Penicillin G	2.8	carboxyl
		Amoxicillin	2.0/7.3	Carboxy/amino
		Piperacillin	2.74/5.13	Carboxyl/pyridine ring
	Cephalosporins	Cefpelin	2.74/5.13	Carboxyl/pyridine ring
		Ceftiofur	2.68	carboxyl
Aminoglycoside antibiotics	Aminoglycosides	gentamicin	7.5–9	amino
		Tobramycin	7.5–9	amino
		Ampramycin	7.5–9	amino
Tetracycline antibiotics	Tetracyclines	tetracycline	2.8–3.4/7.2–7.8/9.1–9.7	Phenolic hydroxyl/enol hydroxyl/dimethylamino
		oxytetracycline	2.8–3.4/7.2–7.8/9.1–9.7	Phenolic hydroxyl/enol hydroxyl/dimethylamino
		aureomycin	2.8–3.4/7.2–7.8/9.1–9.7	Phenolic hydroxyl/enol hydroxyl/dimethylamino
Fluoroquinolone antibiotics	Fluoroquinolones	Ciprofloxacin	3–4/6/7.5–9/10–11	Carboxyl/enol hydroxyl/amino/other groups
		Levofloxacin	3–4/6/7.5–9/10–11	Carboxyl/enol hydroxyl/amino/other groups
		Moxifloxacin	3–4/6/7.5–9/10–11	Carboxyl/enol hydroxyl/amino/other groups
Sulfonamide antibiotics	Sulfonamides	Sulfamethoxazole	2/5–7.5	Sulfonamide/other groups
		Sulfadiazine	2/5–7.5	Sulfonamide/other groups

## Data Availability

Data sharing is not applicable (only appropriate if no new data is generated or the article describes entirely theoretical research).

## References

[1] Yi L., Meng Z., Yang W., Li S., Hu J., Sun W., Ni J. (2024). Profiles, drivers, and prioritization of antibiotics in China’s major rivers. J. Hazard. Mater..

[2] Verma M., Haritash A.K. (2020). Photocatalytic degradation of Amoxicillin in pharmaceutical wastewater: A potential tool to manage residual antibiotics. Environ. Technol. Innov..

[3] Rueda-Marquez J.J., Levchuk I., Ibañez P.F., Sillanpää M. (2020). A critical review on application of photocatalysis for toxicity reduction of real wastewaters. J. Clean. Prod..

[4] Latiful K., David N., Rachadaporn B., Chanthai S., Otgonbayar Z., Oh W.-C. (2024). Photocatalytic removal of pharmaceutical antibiotics induced pollutants by MXene-based composites: Comprehensive review. Sustain. Mater. Technol..

[5] Feng Y., Gong S., Wang Y., Ban C.G., Qu X.L., Ma J.P., Duan Y.Y., Lin C., Yu D.M., Xia L. (2025). Noble-Metal-Free Cocatalysts Reinforcing Hole Consumption for Photocatalytic Hydrogen Evolution with Ultrahigh Apparent Quantum Efficiency. Adv. Mater..

[6] Li Y.-N., Chen Z.-Y., Wang M.-Q., Zhang L.-z., Bao S.-J. (2018). Interface engineered construction of porous g-C_3_N_4_/TiO_2_ heterostructure for enhanced photocatalysis of organic pollutants. Appl. Surf. Sci..

[7] Neeraj S., Stuti T., Mohd S., Kant Choubey R., Singh A. (2021). Study of Optical and Electrical Properties of Graphene Oxide. Mater. Today Proc..

[8] Mamba G., Gangashe G., Moss L., Hariganesh S., Thakur S., Vadivel S., Mishra A.K., Vilakati G.D., Muthuraj V., Nkambule T.T.I. (2020). State of the art on the photocatalytic applications of graphene based nanostructures: From elimination of hazardous pollutants to disinfection and fuel generation. J. Environ. Chem. Eng..

[9] Fu Y., Yao X., Ji X., Zhou L., Tong Y., Zhang R., Wang X. (2024). Defective ordered macroporous ZIF–8/ZnO heterostructure for enhanced visible–light photo–oxidation performance. J. Alloys Compd..

[10] Chen S., Zhang L., Alshammari D.A., Hessien M.M., Yu W., Cui L., Ren J., El-Bahy Z.M., Guo Z. (2025). Z-scheme Ag_2_O/ZnO heterostructure on carbon fibers for efficient photocatalysis of tetracycline. Sep. Purif. Technol..

[11] Lu H., Yang J., Xu J. (2024). Study of the Photocatalytic Degradation of Tetracycline by BiOI/Brookite TiO_2_ Composites. Hans J. Chem. Eng. Technol..

[12] Wang L., Tang M., Wang H., Chen L., Long Z., Gao D. (2023). Study on Photocatalytic Degradation of Tetracycline by Ag_2_CO_3_/ZIF-8/CF Composites. J. Yancheng Inst. Technol. (Nat. Sci. Ed.).

[13] Lerdwiriyanupap T., Waehayee A., Choklap T., Prachanat J., Nakajima H., Chankhanittha T., Butburee T., Siritanon T. (2024). CeO_2_/BiYO_3_ photocatalyst for the degradation of tetracycline under visible light irradiation. Ceram. Int..

[14] Zhao Q., Wang J., Li Z., Guo Y., Tang B., Abudula A., Guan G. (2021). Two-dimensional Ti_3_C_2_T_X_-nanosheets/Cu_2_O composite as a high-performance photocatalyst for decomposition of tetracycline. Carbon Resour. Convers..

[15] Talreja N., Ashfaq M., Chauhan D., Viswanathan M.R. (2023). PVP encapsulated MXene coated on PET surface (PMP)-based photocatalytic materials: A novel photo-responsive assembly for the removal of tetracycline. Environ. Res..

[16] Sun J., Farid M.U., Boey M.W., Sato Y., Chen G., An A.K. (2023). MXene-PVA-TiO_2_-based photothermal-catalytic membrane with high structural stability for efficient desalination and photodegradation. Chem. Eng. J..

[17] Atta M.M., Elbasiony A.M., Henaish A.M.A., Zhang Q. (2024). Enhancement of optical and photocatalytic properties of polyethylene oxide/polyvinylpyrrolidone blend reinforced with Ti_3_C_2_ MXene. Synth. Met..

[18] Naguib M., Mashtalir O., Carle J., Presser V., Lu J., Hultman L., Gogotsi Y., Barsoum M.W. (2012). Two-Dimensional Transition Metal Carbides. ACS Nano.

[19] Sengupta J.A.-O., Hussain C.A.-O. (2025). MXene-Based Electrochemical Biosensors: Advancing Detection Strategies for Biosensing (2020–2024). Biosensors.

[20] Zhu M., Cai C., Lin X., Yang R., Lin X., Li W., Li C., Gao F., Zhang J. (2025). Advances in the application of MXenes and MXene-composites in MXene-based lithium anodes for lithium metal batteries. Mater. Today Chem..

[21] Bu Y., Wang Y., Wu H., Wang H., Wang B., Yang Y., Huang L., Tang J. (2025). Recent advances in flexible piezoresistive pressure sensors based on MXene materials. Mater. Today Chem..

[22] Lin J., Chang C.-W., Chang C.-T., Manibalan K., Tsai T.-H., Lin Y.-C., Hsu Y.-S., Chang M.-H., Chen J.-T. (2025). Wearable and stretchable electronics enabled by MXene–PVA films via layer-by-layer assembly with integrated UV, thermal, and mechanical sensing†. Chem. Commun..

[23] Serafin J., Dziejarski B., Achieng G.O., Vendrell X., Chaitoglou S., Amade-Rovira R. (2025). Comprehensive analysis of MAX phase and MXene materials for advanced photocatalysis, electrocatalysis and adsorption in hydrogen evolution and storage. J. Ind. Eng. Chem..

[24] Carey M., Barsoum M.W. (2021). MXene polymer nanocomposites: A review. Mater. Today Adv..

[25] Hussain S.N., Rao K.R., Ali M.S., Mubarak N.M., Jatoi A.S., Malafaia G., Azad A.K. (2023). MXene as emerging material for photocatalytic degradation of environmental pollutants. Coord. Chem. Rev..

[26] Li K., Zhang S., Li Y., Fan J., Lv K. (2021). MXenes as noble-metal-alternative co-catalysts in photocatalysis. Chin. J. Catal..

[27] Ghanbari R., Zare E.N. (2024). Engineered MXene-polymer composites for water remediation: Promises, challenges and future perspective. Coord. Chem. Rev..

[28] Fatima S., Hakim M.W., Zheng X., Sun Y., Li Z., Han N., Li M., Wang Z., Han L., Wang L. (2025). Constructing nitrogen-doped graphene quantum dots/tantalum carbide MXene heterojunctions as bifunctional catalysts for efficient water splitting. Int. J. Hydrogen Energy.

[29] Wu D., Tong Z., Wang J., Chen G., Zhu A., Tong H., Dang M. (2025). Hole transfer promotion on La-MOF@MXene by valence band structure regulation for the efficient photocatalytic degradation. Sep. Purif. Technol..

[30] Jin P., Han P., Li X., Li K. (2023). Titanium carbide MXenes-initiated plasmonic metal-support interaction for effective photocatalysis on uncoated Ag nanoparticles. Appl. Surf. Sci..

[31] Jian M., Wang Y. (2025). Construction of MXenes/COFs heterojunctions for photo/electrocatalytic applications: Opportunities and challenges. Chem. Eng. J..

[32] Huanhuan S., Panpan Z., Zaichun L., Park S., Lohe M.R., Wu Y., Shaygan Nia A., Yang S., Feng X. (2021). Ambient-Stable Two-Dimensional Titanium Carbide (MXene) Enabled by Iodine Etching. Angew. Chem. Int. Ed..

[33] Wang D., Zhou C., Filatov A.S., Cho W.J., Lagunas F., Wang M.Z., Vaikuntanathan S., Liu C., Klie R.F., Talapin D.V. (2023). Direct synthesis and chemical vapor deposition of 2D carbide and nitride MXenes. Science.

[34] Ahmad M.M., Ma Y., Badshah M., Ali S., Idrees M., Ismail M.A., Khan S., Javed M.S., Tamang T.L., Pu Q. (2024). 2D-MXene: Progress in Synthesis, Intercalation, and Applications in Microfluidic Sensors. Surf. Interfaces.

[35] Sun Y., Sun Y., Meng X., Gao Y., Dall’Agnese Y., Chen G., Dall’Agnese C., Wang X.F. (2019). Eosin Y-sensitized partially oxidized Ti_3_C_2_ MXene for photocatalytic hydrogen evolution. Catal. Sci. Technol..

[36] Singh S., Dharavath S., Kodali S., Dash R.K. (2025). The role of delaminating agents on the structure, morphology, bonding and electrical properties of HF etched MXenes. FlatChem.

[37] Peng C., Yang X., Li Y., Yu H., Wang H.J., Peng F. (2016). Hybrids of Two-Dimensional Ti_3_C_2_ and TiO_2_ Exposing {001} Facets toward Enhanced Photocatalytic Activity. ACS Appl. Mater. Interfaces.

[38] Gentile A., Ferrara C., Tosoni S., Balordi M., Marchionna S., Cernuschi F., Kim M.-H., Lee H.-W., Ruffo R. (2020). Enhanced Functional Properties of Ti_3_C_2_T MXenes as Negative Electrodes in Sodium-Ion Batteries by Chemical Tuning. Small Methods.

[39] Zhang P., Zheng Q., Bashir T., Ali T., Khan S., Alenad A.M., Raza S. (2024). Reliance of MXene terminating groups on various synthetic strategies and its hot electron dynamics at MXene interfaces. J. Environ. Chem. Eng..

[40] Sang X., Xie Y., Lin M.W., Alhabeb M., Van Aken K.L., Gogotsi Y., Kent P.R.C., Xiao K., Unocic R.R. (2016). Atomic Defects in Monolayer Titanium Carbide (Ti_3_C_2_T_x_) MXene. ACS Nano.

[41] Xie X., Zhang N. (2020). Positioning MXenes in the Photocatalysis Landscape: Competitiveness, Challenges, and Future Perspectives. Adv. Funct. Mater..

[42] Miao B., Bashir T., Zhang H., Ali T., Raza S., He D., Bai J. (2024). Impact of various 2D MXene surface terminating groups in energy conversion. Renew. Sustain. Energy Rev..

[43] Kahila B., Ehsan G., Saleem R., Babenko A., Paimard G., Bashir T., Maleki-Ghaleh H., Jie L., Orooji Y. (2024). Recent advancements in MXenes synthesis, properties, and cutting-edge applications: A comprehensive review. J. Environ. Chem. Eng..

[44] Paria E., Aydin H., Stanisław W., Kun-Yi A., Zahra S., Ghanbari F. (2024). Recent advances in design and engineering of MXene-based catalysts for photocatalysis and persulfate-based advanced oxidation processes: A state-of-the-art review. Chem. Eng. J..

[45] Wang S., Du Y.L., Liao W.H. (2017). Tunable band gap and optical properties of surface functionalized Sc_2_C monolayer. Chin. Phys. B.

[46] Hassan N.S., Jalil A.A., Bahari M.B., Izzuddin N.M., Fauzi N.A.F.M., Jusoh N.W.C., Kamaroddin M.F.A., Saravanan R., Tehubijuluw H. (2024). A critical review of MXene-based composites in the adsorptive and photocatalysis of hexavalent chromium removal from industrial wastewater. Environ. Res..

[47] He Y., Chen X., Wu Z., Xue Q., Tian F. (2023). In situ fabrication of N-doped Ti_3_C_2_T_x_-MXene-modified BiOBr Schottky heterojunction with high photoelectron separation efficiency for enhanced photocatalytic ammonia synthesis. J. Alloys Compd..

[48] Liu X., Xu F., Li Z., Liu Z., Yang W., Zhang Y., Yang H.Y. (2022). Design strategy for MXene and metal chalcogenides/oxides hybrids for supercapacitors, secondary batteries and electro/photocatalysis. Coord. Chem. Rev..

[49] Soyoung P., Sewoon K., Yeonji Y., Saravanakumar K., Lee E., Yoon Y., Park C.M. (2023). Adsorptive and photocatalytic performance of cobalt-doped ZnTiO_3_/Ti_3_C_2_T_x_ MXene nanohybrids towards tetracycline: Kinetics and mechanistic insight. J. Hazard. Mater..

[50] Rasheed A., El Sayed M.E., Yousaf S., Samir A., Warsi M.F., El-Bahy Z.M. (2024). Fluorine-doped cobalt ferrite integrated into 2D MXene with extended solar spectrum response and boosted charge separation for the removal of recalcitrant organic pollutants. Results Phys..

[51] Yu X.-f., Cheng J.-b., Liu Z.-b., Li Q.-z., Li W.-z., Yang X., Xiao B. (2015). The band gap modulation of monolayer Ti_2_CO_2_ by strain. RSC Adv..

[52] Ding Y.M., Nie X., Dong H.A.-O., Rujisamphan N., Li Y.A.-O. (2020). Many-body effects in an MXene Ti_2_CO_2_ monolayer modified by tensile strain: GW-BSE calculations. Nanoscale Adv..

[53] Kandemir Z., Torun E., Paleari F., Yelgel C., Sevik C. (2022). Surface termination dependence of electronic and optical properties in Ti_2_CO_2_ MXene monolayers. Phys. Rev. Mater..

[54] Hart J.A.-O., Hantanasirisakul K.A.-O., Lang A.C., Anasori B., Pinto D., Pivak Y., van Omme J.A.-O., May S.J., Gogotsi Y., Taheri M.L. (2019). Control of MXenes’ electronic properties through termination and intercalation. Nat. Commun..

[55] Halim J., Persson I., Eklund P., Persson P.O.Å., Rosen J. (2018). Sodium hydroxide and vacuum annealing modifications of the surface terminations of a Ti_3_C_2_ (MXene) epitaxial thin film. RSC Adv..

[56] Iravani S.A.-O., Varma R.A.-O. (2022). MXene-Based Photocatalysts in Degradation of Organic and Pharmaceutical Pollutants. Molecules.

[57] Irfan S., Khan S.A.-O., Din M.A.U., Dong F., Chen D. (2023). Retrospective on Exploring MXene-Based Nanomaterials: Photocatalytic Applications. Molecules.

[58] He X., Kai T., Ding P.A.-O. (2021). Heterojunction photocatalysts for degradation of the tetracycline antibiotic: A review. Environ. Chem. Lett..

[59] Wang Y., Tan G., Feng S., Liu Y., Liu W., Xia A., Ren H., Lv L. (2023). Preparation of exposed (001) facets TiO_2_/Ti_3_C_2_ MXene and performance of near infrared light photodegradation. Ceram. Int..

[60] Lai C., Yan B., Yuan R., Chen D., Wang X., Wang M., He H., Tu J.A.-O. (2023). In situ growth of TiO_2_/Ti_3_C_2_ MXene Schottky heterojunction as a highly sensitive photoelectrochemical biosensor for DNA detection. RSC Adv..

[61] Ding W., Deng H., Guo S., Li L., Wang P., Zhu H., Hao D., Li C., Tang X., Wang Q. (2025). MXene-based composites for adsorption and photocatalytic immobilization of U(VI): Current progress and future perspectives. Sep. Purif. Technol..

[62] ZHENG Z.X., WANG E.H., HOU X.M., YANG T. (2022). University of Science and Technology Beijing. The stability and improvement of two-dimensional transition metal carbides and/or carbonitrides (MXene). J. Eng. Sci..

[63] Zhu H., Liang Z., Xue S., Ren X., Liang X., Xiong W., Gao L., Liu A. (2022). DFT practice in MXene-based materials for electrocatalysis and energy storage: From basics to applications. Ceram. Int..

[64] Khazaei M., Arai M., Sasaki T., Chung C.-Y., Venkataramanan N.S., Estili M., Sakka Y., Kawazoe Y. (2013). Novel Electronic and Magnetic Properties of Two-Dimensional Transition Metal Carbides and Nitrides. Adv. Funct. Mater..

[65] Wang Q., Xie H., Nie Y.-H., Ren W. (2013). Enhancement of thermoelectric efficiency in triple quantum dots by the Dicke effect. Phys. Rev. B.

[66] Shein I.R., Ivanovskii A.L. (2012). Graphene-like titanium carbides and nitrides Ti_n+1_C_n_, Ti_n+1_N_n_ (n = 1, 2, and 3) from de-intercalated MAX phases: First-principles probing of their structural, electronic properties and relative stability. Comput. Mater. Sci..

[67] Low J., Zhang L., Tong T., Shen B., Yu J. (2018). TiO_2_/MXene Ti_3_C_2_ composite with excellent photocatalytic CO_2_ reduction activity. J. Catal..

[68] Li J., Peng H., Luo B., Cao J., Ma L., Jing D. (2023). The enhanced photocatalytic and photothermal effects of Ti_3_C_2_ Mxene quantum dot/macroscopic porous graphitic carbon nitride heterojunction for Hydrogen Production. J. Colloid Interface Sci..

[69] Oyehan T.A., Salami B.A., Abdulrasheed A.A., Hambali H.U., Gbadamosi A., Valsami-Jones E., Saleh T.A. (2023). MXenes: Synthesis, properties, and applications for sustainable energy and environment. Appl. Mater. Today.

[70] Xin L., Guohong W., Qiang L., Wang Y., Lu X. (2023). Dual optimized Ti_3_C_2_T_x_ MXene@ZnIn2S4 heterostructure based on interface and vacancy engineering for improving electromagnetic absorption. Chem. Eng. J..

[71] Tao C., Longlu W., Yutang L., Zhang S., Dong W., Chen H., Yi X., Yuan J., Xia X., Liu C. (2018). Ag_3_PO_4_/Ti_3_C_2_ MXene interface materials as a Schottky catalyst with enhanced photocatalytic activities and anti-photocorrosion performance. Appl. Catal. B Environ..

[72] Zeng W., Ye X., Dong Y., Zhang Y., Sun C., Zhang T., Guan X., Guo L. (2024). MXene for photocatalysis and photothermal conversion: Synthesis, physicochemical properties, and applications. Coord. Chem. Rev..

[73] Wang H., Li X., Zhao X., Li C., Song X., Zhang P., Huo P. (2022). A review on heterogeneous photocatalysis for environmental remediation: From semiconductors to modification strategies. Chin. J. Catal..

[74] Meng X., Wang L., Wang X., Zhen M., Hu Z., Guo S.Q., Shen B. (2023). Recent developments and perspectives of MXene-Based heterostructures in photocatalysis. Chemosphere.

[75] Li Z., Xiong J., Song H., Liu S., Huang Y., Huang Y., Luo X. (2024). Synergistically enhancing CO_2_ adsorption/activation and electron transfer in ZIF-67/Ti_3_C_2_T_x_ MXene for boosting photocatalytic CO_2_ reduction. Sep. Purif. Technol..

[76] Khesali A.S., Mahdiyeh Z., Saeid A., Fooladloo M.A. (2023). Investigation of the optical and electronic properties of functionalized Ti_3_C_2_ Mxene with halid atoms using DFT calculation. Mater. Today Commun..

[77] Yao X., Yan-Fang Z., Wei X., Wu Z.-G., Li Y.-C., Lai J., Li S., Wang E., Yang Z.-G., Xu C.-L. (2020). Deciphering an Abnormal Layered-Tunnel Heterostructure Induced by Chemical Substitution for the Sodium Oxide Cathode. Angew. Chem. Int. Ed..

[78] Shi A., Sun D., Zhang X., Ji S., Wang L., Li X.A., Niu X. (2022). Direct Z-Scheme Photocatalytic System: Insights into the Formative Factors of Photogenerated Carriers Transfer Channel from Ultrafast Dynamics. ACS Catal..

[79] Huang X., Yin K., Zhang S., Wu T., Yuan Y., Wang X., Wang D. (2023). Interfacial chemical bond-modulated Z-scheme Cs_2_AgBiBr_6_/WO_3_ enables stable and highly efficient photocatalysis. Appl. Surf. Sci..

[80] Yuan H., Xiao W., Zhang X., Bao J., Li W., Huang B., He G. (2024). Ti_3_C_2_ MXene flakes assisted Mn_0.5_Cd_0.5_S/Ti_3_C_2_ MXene/g-C_3_N_4_ Z-scheme heterojunction for enhancing photocatalytic hydrogen evolution. J. Power Sources.

[81] Zhou Y., Yu M., Liang H., Chen J., Xu L., Niu J. (2021). Novel dual-effective Z-scheme heterojunction with g-C_3_N_4_, Ti_3_C_2_ MXene and black phosphorus for improving visible light-induced degradation of ciprofloxacin. Appl. Catal. B Environ..

[82] Karunamoorthy S., Keunyoung Y., Velusamy M., Yea Y., Jagan G., Park C.M. (2023). Construction of novel In_2_S_3_/Ti_3_C_2_ MXene quantum dots/SmFeO_3_ Z-scheme heterojunctions for efficient photocatalytic removal of sulfamethoxazole and 4-chlorophenol: Degradation pathways and mechanism insights. Chem. Eng. J..

[83] Liu Y., Li Y.-H., Li X., Zhang Q., Yu H., Peng X., Peng F. (2020). Regulating Electron–Hole Separation to Promote Photocatalytic H2 Evolution Activity of Nanoconfined Ru/MXene/TiO_2_ Catalysts. ACS Nano.

[84] Soni V., Singh P., Phan Quang H.H., Parwaz Khan A.A., Bajpai A., Van Le Q., Thakur V.K., Thakur S., Nguyen V.-H., Raizada P. (2022). Emerging architecture titanium carbide (Ti_3_C_2_T_x_) MXene based photocatalyst toward degradation of hazardous pollutants: Recent progress and perspectives. Chemosphere.

[85] Peng C., Zhou T., Wei P., Xu W., Pan H., Peng F., Jia J., Zhang K., Yu H. (2021). Photocatalysis over MXene-based hybrids: Synthesis, surface chemistry, and interfacial charge kinetics. APL Mater..

[86] Chaudhuri K., Alhabeb M., Wang Z., Shalaev V.M., Gogotsi Y., Boltasseva A. (2018). Highly Broadband Absorber Using Plasmonic Titanium Carbide (MXene). ACS Photonics.

[87] Li C., Kan C., Meng X., Liu M., Shang Q., Yang Y., Wang Y., Cui X. (2022). Self-Assembly 2D Ti_3_C_2_/g-C_3_N_4_ MXene Heterojunction for Highly Efficient Photocatalytic Degradation of Tetracycline in Visible Wavelength Range. Nanomaterials.

[88] Nasri M.S., Samsudin M.F., Tahir A.A., Sufian S. (2022). Effect of MXene Loaded on g-C_3_N_4_ Photocatalyst for the Photocatalytic Degradation of Methylene Blue. Energies.

[89] Diao Y., Yan M., Li X., Zhou C., Peng B., Chen H., Zhang H. (2020). In-situ grown of g-C_3_N_4_/Ti_3_C_2_/TiO_2_ nanotube arrays on Ti meshes for efficient degradation of organic pollutants under visible light irradiation. Colloids Surf. A Physicochem. Eng. Asp..

[90] Hu X., Li G., Zhang Y., Lu M., Pu W., Dai Y., Wang H. (2024). A novel iron oxide (Fe_3_O_4_)-laden titanium carbide (Ti_3_C_2_) MXene stacks for the efficient removal of tetracycline from aqueous solution. Chemosphere.

[91] Xu Q., Wang P., Wang Z., Shen J., Han X., Zheng X., Song K. (2022). Aerosol self-assembly synthesis of g-C_3_N_4_/MXene/Ag_3_PO_4_ heterostructure for enhanced photocatalytic degradation of tetracycline hydrochloride. Colloids Surf. A Physicochem. Eng. Asp..

[92] Cai J., Peng Y., Hu P., Zhou Y., Yao Q., Lyu H., Gao Y. (2025). H_2_O_2_-assisted room temperature preparation of crystalline TiO_2_ and Ti_3_C_2_T_x_-derived C-doped amorphous TiOx homojunction for photocatalytic degradation of tetracycline. Appl. Surf. Sci..

[93] Amari A., Aljibori H.S.S., Ismail M.A., Diab M.A., El-Sabban H.A., Umarov A., Madaminov S., Elboughdiri N. (2025). Engineering novel 2D MXene-based dual Z-scheme heterojunction photocatalyst for enhanced TC hydrochloride degradation and hydrogen evolution. J. Water Process Eng..

[94] Xu M., Peng M., Tang H., Zhou W., Qiao B., Ma D. (2024). Renaissance of Strong Metal–Support Interactions. J. Am. Chem. Soc..

[95] Yang Z., Yang L., Liu Y., Chen L. (2024). Photocatalytic Deposition of Au Nanoparticles on Ti_3_C_2_T_x_ MXene Substrates for Surface-Enhanced Raman Scattering. Molecules.

[96] Chen H., Yang Z., Wang X., Polo-Garzon F., Halstenberg P.W., Wang T., Suo X., Yang S.-Z., Meyer H.M., Wu Z. (2021). Photoinduced Strong Metal–Support Interaction for Enhanced Catalysis. J. Am. Chem. Soc..

[97] Wu Y., Li J., Sui G., Chai D.F., Li Y., Guo D., Chu D., Liang K. (2024). Interface and doping engineering of V2C-MXene-based electrocatalysts for enhanced electrocatalysis of overall water splitting. Carbon Energy.

[98] Zhou Q., Hong P., Shi X., Li Y., Yao K., Zhang W., Kong L. (2023). Efficient degradation of tetracycline by a novel nanoconfinement structure Cu_2_O/Cu@ MXene composite. J. Hazard. Mater..

[99] Zheng Z., Liu D., Sun X., Geng Z., Wang Q., Hou J. (2025). SILAR synthesis of CdS-ZnS/TiO_2_ NTs for photocatalytic H2 evolution and dye degradation. Colloids Surf. A Physicochem. Eng. Asp..

[100] Xiao R., Zhao C., Zou Z., Chen Z., Tian L., Xu H., Yang X. (2020). In situ fabrication of 1D CdS nanorod/2D Ti_3_C_2_ MXene nanosheet Schottky heterojunction toward enhanced photocatalytic hydrogen evolution. Appl. Catal. B Environ..

[101] Chen Y., Zhong W., Chen F., Wang P., Fan J., Yu H. (2022). Photoinduced self-stability mechanism of CdS photocatalyst: The dependence of photocorrosion and H2-evolution performance. J. Mater. Sci. Technol..

[102] Tan Q., Yu Z., Chen Y., He N. (2024). Mixed-valent FeWO_4_-coated 2D Ti_3_C_2_ MXene photocatalysts for photo-fenton removal of many common pollutants in water. Ceram. Int..

[103] Kashif R., Mohamed H., Adnan A., Ren C.E., Gogotsi Y., Mahmoud K.A. (2016). Antibacterial Activity of Ti_3_C_2_T_x_ MXene. ACS Nano.

[104] Gao K., Hou L.A., An X., Huang D., Yang Y. (2023). BiOBr/MXene/gC_3_N_4_ Z-scheme heterostructure photocatalysts mediated by oxygen vacancies and MXene quantum dots for tetracycline degradation: Process, mechanism and toxicity analysis. Appl. Catal. B Environ..

[105] Wilson R.B., Coh S. (2020). Parametric dependence of hot electron relaxation timescales on electron-electron and electron-phonon interaction strengths. Commun. Phys..

[106] Ostovar B.A.-O., Lee S.A.-O.X., Mehmood A.A.-O., Farrell K.A.-O., Searles E.A.-O., Bourgeois B.A.-O.X., Chiang W.A.-O., Misiura A., Gross N.A.-O., Al-Zubeidi A. (2024). The role of the plasmon in interfacial charge transfer. Sci. Adv..

[107] Liu J., Yang J., Zhu G., Li J., Li Y., Zhai Y., Song H., Yang Y., Li H. (2024). Revealing the Ultrafast Energy Transfer Pathways in Energetic Materials: Time-Dependent and Quantum State-Resolved. JACS Au.

[108] Mir S.H., Yadav V.K., Singh J.K. (2020). Recent Advances in the Carrier Mobility of Two-Dimensional Materials: A Theoretical Perspective. ACS Omega.

[109] Shin H.A.-O., Jeong W., Han T.A.-O. (2024). Maximizing light-to-heat conversion of Ti_3_C_2_T_x_ MXene metamaterials with wrinkled surfaces for artificial actuators. Nat. Commun..

[110] Wang T., Yao C., Gao R., Holicky M., Hu B., Liu S., Wu S., Kim H., Ning H., Torrisi F. (2024). Ultrafast Carrier and Lattice Cooling in Ti_2_CT_x_ MXene Thin Films. Nano Lett..

[111] Chang L., Huan Z., Qinghan W., Wu J., Chen P., Song S., Zhan C., Wang L., Jia F. (2024). Efficient gold recovery from low concentrated Au(S_2_O_3_)_23_—Solution through enhanced photocatalysis via photothermal and surface plasmon resonance assistance on MoS_2_/MXene/Ag. Desalination.

[112] Wang D., Fang Y., Yu W., Wang L., Xie H., Yue Y. (2021). Significant solar energy absorption of MXene Ti_3_C_2_T_x_ nanofluids via localized surface plasmon resonance. Sol. Energy Mater. Sol. Cells.

[113] Niu X., Lu X., He B., Xiao X., Wang S., Liang Z., Ma L.F. (2025). Dual electron transfer path and Ti_3_C_2_T_x_ LSPR photothermal enhancement in 2D/2D/2D Ti_3_C_2_T_x_ MXene@TiO_2_/CdIn_2_S_4_ ternary heterojunction for enhanced photocatalytic H_2_ production: Construction, kinetics, and mechanistic insights. Appl. Catal. B Environ. Energy.

[114] Liu W., Sun M., Ding Z., Gao B., Ding W. (2021). Ti_3_C_2_ MXene embellished g-C_3_N_4_ nanosheets for improving photocatalytic redox capacity. J. Alloys Compd..

[115] Wang A., Wen Y., Zhu H., Liu Z., Wang H., Yao W., Lin H. (2024). Oxygen vacancy mediated photocatalysis of Ti_3_C_2_ MXene quantum dots/W_18_O_49_ hybrid membrane for peroxymonosulfate-enhanced oxidation degradation. Chem. Eng. J..

[116] Ling Z., Lu T., Zhenxi Y., Xu B., Chen W., Tang Y., Li L., Wang J. (2023). Engineering of Bi_2_O_2_CO_3_/Ti_3_C_2_T_x_ heterojunctions co-embedded with surface and interface oxygen vacancies for boosted photocatalytic degradation of levofloxacin. Chem. Eng. J..

[117] Ma Y., Xu D., Chen W., Tang Y., Wang X., Li L., Wang J. (2022). Oxygen-vacancy-embedded 2D/2D NiFe-LDH/MXene Schottky heterojunction for boosted photodegradation of norfloxacin. Appl. Surf. Sci..

[118] Yu M., Liang H., Zhan R., Xu L., Niu J. (2021). Sm-doped g-C_3_N_4_/Ti_3_C_2_ MXene heterojunction for visible-light photocatalytic degradation of ciprofloxacin. Chin. Chem. Lett..

[119] Fatima S., Anwar M., Almalki A.S.A., Alhadhrami A., Warsi M.F., El-Bahy Z.M. (2024). Fabrication of rare earth (Tb^+3^) and alkaline earth metal (Mg^+2^) Co-doped CdA_l2_O_4_@MXene composite: A unique approach to tune bandgap energy through quantum confinement effect for photocatalytic applications. Ceram. Int..

[120] Abbas H.A., Abbas K.K., Al-Ghaban A.M.A. (2025). Magnetic MXene/g-C_3_N_4_ nano catalyst for photocatalytic degradation of clindamycin contaminate in wastewater. Results Chem..

[121] Yan C., Zongxue Y., Guangcheng Y., Tan Q., He N., Guo S., Xia S., Chen Z. (2024). Interlayered double-doped N,P-MXene/ZnIn_2_S_4_ Schottky junction composite photocatalyst: Efficient removal of ciprofloxacin and methyl orange from complex wastewater. J. Alloys Compd..

[122] Li R., Chen A., Deng Q., Zhong Y., Kong L., Yang R. (2022). Well-designed MXene-derived Carbon-doped TiO_2_ coupled porous g-C_3_N_4_ to enhance the degradation of ciprofloxacin hydrochloride under visible light irradiation. Sep. Purif. Technol..

[123] Hussain M., Mahmoud M.H.H., Rasheed A., El Azab I.H., Anwar M., El-Bahy Z.M. (2024). Silver-doped cadmium aluminate and its MXene based composite for visible-light driven photocatalytic degradation of organic pollutants. Opt. Mater..

[124] Valli K.P., Kala S.M.J., Selvam V., Anitha C., Malathi B., Prakash K.S., Karutha Pandian S. (2024). Novel hierarchical nanocomposites of g-C_3_N_4_/MXene-Sm_2_O_3_ for enhanced cefixime degradation under visible light. J. Phys. Chem. Solids.

[125] Li D., Wang G., Ye Y., Boutinaud P., Zheng X., Xu J., Kang F. (2024). Recent advances in the synthesis, photo-/electrocatalytic properties and applications of MXenes/bismuth-related composites. Chem. Eng. J..

[126] Liu Z., Yu X., Wang K., Wei Y., Zhang J., Niu J. (2025). Exploiting Cu_2_O-MXene(Ti_3_C_2_) photocatalysts with visible light drive for the degradation of sulfamethazine in the environment. Opt. Mater..

[127] Dan L., Jiajie X., Changkun L. (2023). Constructing MXene-derived Z-Scheme g-C_3_N_4_/Ti_3_C_2_T_X_/Ag_3_PO_4_ photocatalysts with enhanced charge transfer for aquatic organic pollutants removal. Colloids Surf. A Physicochem. Eng. Asp..

[128] Zhang S., Wang Y., Mersal G.A., Alhadhrami A., Alshammari D.A., Wang Y., Algadi H., Song H. (2024). Enhanced photocatalytic CO_2_ reduction via MXene synergism: Constructing an efficient heterojunction structure of g-C_3_N_4_/Nb_2_C/CsPbBr_3_. Adv. Compos. Hybrid Mater..

[129] Xu R., Wei G., Xie Z., Diao S., Wen J., Tang T., Hu G. (2024). V_2_C MXene–modified g-C_3_N_4_ for enhanced visible-light photocatalytic activity. J. Alloys Compd..

[130] Subha N., Nagappagari L.R., Sankar A.R. (2024). A review on recent advances in g-C_3_N_4_-MXene nanocomposites for photocatalytic applications. Nanotechnology.

[131] Mousavi S.M., Mohtaram M.S., Rasouli K., Mohtaram S., Rajabi H., Sabbaghi S. (2025). Efficient visible-light-driven photocatalytic degradation of antibiotics in water by MXene-derived TiO_2_-supported SiO_2_/Ti_3_C_2_ composites: Optimisation, mechanism and toxicity evaluation. Environ. Pollut..

[132] Yang J., Yin H., Du A., Tebyetekerwa M., Bie C., Wang Z., Zhang X. (2025). Unveiling O_2_ adsorption on non-metallic active site for selective photocatalytic H_2_O_2_ production. Appl. Catal. B Environ. Energy.

[133] Jie W., Bingyan Y., Keru H., Ji Y., Li X., Li C., Yao C., Cai Z. (2025). pH-responsive fiber membrane with high flux and photocatalytic performance for oily wastewater. J. Environ. Chem. Eng..

[134] Zhenhai W.A.N.G., Zikai Z.H.O.U., Sen W.A.N.G., Zhi F.A.N.G. (2024). Enhanced degradation of tetracycline by gas-liquid discharge plasma coupled with g-C_3_N_4_/TiO_2_. Plasma Sci. Technol..

[135] Mirzaei R., Yunesian M., Nasseri S., Gholami M., Jalilzadeh E., Shoeibi S., Bidshahi H.S., Mesdaghinia A.A.-O. (2017). An optimized SPE-LC-MS/MS method for antibiotics residue analysis in ground, surface and treated water samples by response surface methodology-central composite design. J. Environ. Health Sci. Eng..

[136] Rusu A.A.-O., Buta E.A.-O. (2021). The Development of Third-Generation Tetracycline Antibiotics and New Perspectives. Pharmaceutics.

[137] Eslaminejad S., Rahimi R., Fayazi M. (2025). Green decoration of Pd nanoparticles on MXene/metal organic framework support for photocatalytic degradation of ofloxacin. J. Ind. Eng. Chem..

[138] Zhang H., Bao L., Pan Y., Du J., Wang W. (2024). Interface reconstruction of MXene-Ti_3_C_2_ doped CeO_2_ nanorods for remarked photocatalytic ammonia synthesis. J. Colloid Interface Sci..

[139] Wei C., Junli N., Ye G., Gao C., Wang M., Wang W., Lu X., Ma X., Zhong P. (2024). A review of how to improve Ti_3_C_2_T_x_ MXene stability. Chem. Eng. J..

